# Tet1 deficiency exacerbates oxidative stress in acute kidney injury by regulating superoxide dismutase

**DOI:** 10.7150/thno.87416

**Published:** 2023-09-25

**Authors:** Yu Fan, Yangmian Yuan, Mingrui Xiong, Muchuan Jin, Donge Zhang, Dong Yang, Chengyu Liu, Robert B. Petersen, Kun Huang, Anlin Peng, Ling Zheng

**Affiliations:** 1 Hubei Key Laboratory of Cell Homeostasis, Frontier Science Center for Immunology and Metabolism, College of Life Sciences, Wuhan University, Wuhan, China, 430072.; 2 School of Pharmacy, Tongji Medical College and State Key Laboratory for Diagnosis and Treatment of Severe Zoonotic Infectious Diseases, Huazhong University of Science and Technology, Wuhan, China, 430030.; 3 Department of Pharmacy, The Third Hospital of Wuhan and Tongren Hospital of Wuhan University, Wuhan, China, 430070.; 4 Department of Transfusion Medicine, Wuhan Hospital of Traditional Chinese and Western Medicine, Tongji Medical College, Huazhong University of Science and Technology, Wuhan, China, 430030.; 5 Foundational Sciences, Central Michigan University College of Medicine, Mount Pleasant, MI, USA, 48858.

**Keywords:** acute kidney injury, Tet1, DNA methylation, oxidative stress, superoxide dismutase

## Abstract

**Rationale:** Increased methylation of key genes has been observed in kidney diseases, suggesting that the ten-eleven translocation (Tet) methyl-cytosine dioxygenase family as well as 5mC oxidation may play important roles. As a member of the Tet family, the role of Tet1 in acute kidney injury (AKI) remains unclear.

**Methods:** *Tet1* knockout mice, with or without tempol treatment, a scavenger of reactive oxygen species (ROS), were challenged with ischemia and reperfusion (I/R) injury or unilateral ureteral obstruction (UUO) injury. RNA-sequencing, Western blotting, qRT-PCR, bisulfite sequencing, chromatin immunoprecipitation, immunohistochemical staining, and dot blot assays were performed.

**Results:** Tet1 expression was rapidly upregulated following I/R or UUO injury. Moreover, *Tet1* knockout mice showed increased renal injury and renal cell death, increased ROS accumulation, G2/M cell cycle arrest, inflammation, and fibrosis. Severe renal damage in injured *Tet1* knockout mice was alleviated by tempol treatment. Mechanistically, Tet1 reduced the 5mC levels in an enzymatic activity-dependent manner on the promoters of *Sod1* and *Sod2* to promote their expression, thus lowering injury-induced excessive ROS and reducing I/R or UUO injury.

**Conclusions:** Tet1 plays an important role in the development of AKI by promoting SOD expression through a DNA demethylase-dependent mechanism.

## Introduction

Acute kidney injury (AKI) is defined as a rapid decrease in renal function characterized by increased serum creatinine, decreased urine output, or both 1. AKI is associated with high mortality in the absence of effective treatment, and its prevalence in patients admitted to the intensive care unit (ICU) exceeds 50% 1, 2. Moreover, AKI is also a major risk factor for the development of chronic kidney disease (CKD) and end-stage renal disease (ESRD) 1, 3.

Several clinical events contribute to the development of AKI, including renal ischemia-reperfusion (I/R) injury and obstructive nephropathy. Renal I/R injury often occurs during cardiac surgeries, shock, and kidney transplantation 4, 5. The pathogenesis of renal I/R injury is complicated, however, production of excessive reactive oxygen species (ROS) during reperfusion is a major contributor to the injury 6. Excessive ROS contributes to oxidative stress, apoptosis/necrosis, and inflammation 7, 8, which lead to maladaptive cellular responses including cell cycle arrest, metabolic reprogramming, and secretion of profibrotic factors that accelerate the progression of AKI to CKD 9. Obstructive nephropathy is another common clinical event that may cause AKI or CKD 10, 11. In rodent models, unilateral ureteral obstruction (UUO) mimics obstructive nephropathy with development of severe tubular injury, interstitial inflammation and renal fibrosis, serving as a model of irreversible AKI 11. Like I/R injury, excessive ROS production and enhanced oxidative stress also contribute to the pathology associated with UUO 12.

Dynamic regulation of cytosine methylation/demethylation is a common epigenetic modification that regulates disease processes in a cell-type, context-dependent manner 13, 14. The Ten-eleven translocation cytosine dioxygenases (Tets) family, including Tet1, Tet2, and Tet3, are key enzymes that convert 5-methylcytosine (5mC) to 5-hydroxymethylcytosine (5hmC) via an α-ketoglutarate (α-KG) and Fe^2+^ dependent mechanism 15, 16. We recently reported that Tet1 affects the methylation level of the promoters of lipolysis and lipid oxidation-related genes, resulting in an obesity-prone phenotype in *Tet1* insufficient mice fed a high fat diet (HFD) 17. Although an increased Tet1 level has recently been found in the kidneys of CKD patients 18, the exact role of Tet1 in AKI and the following transition to CKD remains unclear.

In the current study of I/R- or UUO-induced renal injury, we found an increased protein level of Tet1 in the kidney, while knockout of *Tet1* aggravated AKI and AKI-CKD transition as suggested by increased inflammation, apoptosis, oxidative stress and fibrosis. RNA-sequencing analysis demonstrated that the superoxide dismutase (SOD) family was downregulated in injured *Tet1* knockout mice. Mechanistic studies demonstrated that *Tet1* knockout affected *Sod1* and *Sod2* expression by increasing the methylation levels on their promoters, thus decreasing their expression. Moreover, treating with tempol, an SOD mimic and strong ROS scavenger, eliminated the worst outcomes observed in injured *Tet1* KO mice. Together, we revealed a critical role for Tet1 in both AKI and AKI-CKD transition stages.

## Materials and Methods

### Animals

Breeding pairs of Tet1^tm1.1Jae^ (JAX #017358, *Tet1*^+/-^) mice were kindly provided by Dr. Wuhan Xiao (Institute of Hydrobiology, CAS) 19. Genotyping to identify wildtype (WT; *Tet1*^+/+^) and knockout (KO; *Tet1*^-/-^) mice was performed as described 17. Both males and females weighing 22 ± 2 g were used in the present study. Animals were handled according to the Guidelines of the China Animal Welfare Legislation, as approved by the Committee on Ethics in the Care and Use of Laboratory Animals, College of Life Sciences, Wuhan University.

### AKI models and treatments

Ischemic AKI was created using a unilateral renal I/R injury model as previously described to reduce surgery caused related mortality 20. Briefly, mice were anesthetized and underwent midline abdominal incisions with the left renal pedicle bluntly clamped for 45 minutes; reperfusion was achieved by removing the clamp. Mice were euthanized at day 3, 7, or 21 to harvest kidneys. For the UUO model, the left ureter of the mouse was ligated at proximal and distal points and then cut between the ligated points 21. Mice were sacrificed 7 days later and kidneys were collected. For folic acid-induced AKI, folic acid (250 mg/kg body-weight; Aladdin, Shanghai, China) was dissolved in NaHCO_3_ (300 mmol/L) and injected intraperitoneally once 22; kidneys were collected at day 1, 3 or 7 after the injection. As previously reported 23, 24, after I/R or UUO injury, tempol (50 mg/kg body-weight; MedChem Express, NJ) was administered by oral gavage once per day for 3 or 7 days.

### Cell culture, plasmids, *in vitro* Hypoxia/Reperfusion (HR) injury and treatment

The mouse renal tubular epithelial cell line TCMK-1 (Otwo Biotech., Guangzhou, China) was cultured in DMEM media (Cytiva, South Logan, UT) plus 10% FBS (Lonsera, shuangru Biotech., China). pCMV-m*Tet1* (full-length mouse *Tet1*) plasmid was a kind gift from Dr. Wuhan Xiao; pCMV-m*Tet1*△CD (mouse *Tet1* enzymatic domain truncation) plasmid was constructed using a standard protocol. TCMK-1 cells were transfected with different plasmids, *in vitro* hypoxia and reperfusion (HR) experiments (12 hr hypoxia followed by 1 hr reperfusion) were performed as previously described 25. For TGF-β recombinant protein (Novoprotein, Suzhou, China) treatment, TCMK-1 cells were treated with 0, 5 or 10 ng/ml TGF-β for 24 h.

### MTT assays

TCMK1 cells were cultured at a density of 3000 cells/well in a 96-well plate for 12 hours, and then transfected with the empty vector, pCMV-m*Tet1*, or pCMV-m*Tet1*△CD. 24 hours later, cells were treated to produce HR injury, then MTT assays were performed as previously reported 26.

### Measurement of SOD activity

A superoxide dismutase (SOD) assay kit (Beyotime Biotech., Shanghai, China) was used to measure the oxidative stress level in the kidneys and TCMK1 cells.

### Assessment of renal function

Blood samples were centrifuged at 2000 g for 15 min at 4 ℃, and serum was collected subsequently. Serum creatinine (Crea) and blood urea nitrogen (BUN) levels were measured using a creatinine reagent kit or a BUN reagent kit (both from Jiancheng Bio., Nanjing, China), respectively.

### Histology, immunohistochemical, immunofluorescent and dihydroethidium staining

Paraffin embedded renal sections were used for hematoxylin-eosin (H&E), sirius red or immunohistochemical staining. H&E staining was performed to evaluate pathological lesions as previously described 27, 28. Sirius red staining (SenBeijia Biotech., Nanjing, China) was performed to detect fibrosis. For immunohistochemical staining, primary antibodies for Ly6G, CD3, F4/80, 5hmC or Tet1 were applied to sections (detailed information in Table S1) overnight at 4 °C, then incubated with an appropriate biotinylated secondary antibody and ABC-peroxidase solution (Vector Laboratories, Burlingame, CA) sequentially, and finally visualized using 3,3′-diaminobenzidine (DAB, Cwbiotech, Beijing, China).

OCT (Sakura Finetek, Torrance, CA) embedded renal cryosections were used for immunofluorescent staining, as well as for renal tubular markers and dihydroethidium (DHE) staining. Cryosections were incubated overnight with a Tet1 antibody (Table S1) followed by an appropriate Alexa Fluor labelled secondary antibody (Thermo Fisher, Waltham, MA). For renal tubular staining, cryosections were incubated with PNA (peanut agglutinin, detecting distal tubules and collecting ducts), or LTL (lotus tetragonolobus lectin, detecting proximal tubules), or DBA (dolichos biflorus agglutinin, detecting collecting ducts) (all from Vector Laboratories), respectively, as previously reported 27, 29. For DHE staining, cryosections were incubated with 10 μM DHE (Beyotime) at 37 °C for 30 min. Sections were then covered with DAPI (Sigma) and antifading medium (Invitrogen). Sections were imaged using a Leica TCS SP8 confocal microscope (Milan, Italy). Quantitative analysis of positively stained cells was performed as previously reported 29, 30.

### Dot blot assays

Genomic DNA was isolated using a genomic DNA extraction kit (Tiangen Biotech., Beijing, China), and dot blot assays were performed 17. Briefly, 100 ng of denatured DNA was spotted onto a nitrocellulose membrane (Bio-Rad, Hercules, CA). After cross-linking and blocking, anti-5hmC or anti-5mC antibody (Active motif, California, CA) was applied, then probed with HRP-conjugated secondary antibody (Bio-Rad), visualized by enhanced chemiluminescence reagent (Beyotime), and quantitated using Quantity One (Bio-Rad).

### Bisulfite sequencing

Bisulfite modification of genomic DNA was performed using a Bisulfite Conversion Kit (Active Motif) following the manufacturer's instruction. The promotors (-1500 bp to +200 bp relative to the +1 transcription start site) of *Sod1* or *Sod2* were amplified using primers designed using MethPrimer (http://www.urogene.org/) (Table S2). PCR was performed using Hot Start Taq Polymerase (Qiagen, Germany). PCR fragments purified from agarose gels were cloned into the pMD18-T vector (Tsking Biotech., Beijing, China). At least 10 clones of each sample were selected for sequencing and analyzed using DNAMAN software (San Ramon, CA) 17.

### RNA sequencing and analysis

Total renal RNA was isolated. RNA sequencing and data analyses were performed by Novogene Bioinformatics (Beijing, China) 31. Differentially expressed genes were assessed with a threshold of adjusted *P* value < 0.05 and |log_2_FoldChange| > 0.5. Novogene's cloud platform (https://magic.novogene.com/) was used for KEGG pathway enrichment, while Graphpad Prism (v8.0.1) was used to generate a heatmap.

### Co-immunoprecipitation (Co-IP)

Renal tissues were ground in immunoprecipitation buffer (Beyotime) supplemented with protease inhibitors and then centrifuged at 8000 g for 10 mins to obtain whole cell lysates. Lysates were immunoprecipitated with the Tet1 antibody or respective IgG with Protein A/G magnetic beads (MedChem Express, NJ) overnight at 4 °C 32. After washing, the beads were boiled in loading buffer and subjected to Western blots.

### Chromatin immunoprecipitation (ChIP)

ChIP assays were performed as previously described 33, 34. Briefly, 48 hours after transfection with empty vector or the pCMV-*mTet1* plasmid, TCMK1 cells were crosslinked with 1% formaldehyde, then quenched with glycine. Crosslinked chromatin was sheared using micrococcal nuclease (New England Biolabs, Beverly, MA) and sonication. Chromatin was immunoprecipitated using anti-Tet1 antibody or rabbit IgG (Table S1). Purified DNA was detected by qPCR (primer sequences provided in Table S2). Input samples were used as the internal control for comparison between samples.

### Western blots and Quantitative real-time PCR (qPCR)

Western blots and qPCR were performed as previously described 35, 36. Antibodies and primers are shown in Tables S1 and S2.

### Statistical analysis

The data are presented as means ± SD. The normal distribution of data was tested using Graphpad Prism (v8.0.1). For two-group comparison, statistical significance of normally distributed data was analyzed using an unpaired two-tailed Student's test, while non-normally distributed data was analyzed using Mann-Whitney U test. For multiple-group comparison of normally distributed data, one-way ANOVA with Tukey's test was used for a single independent variable, while two-way ANOVA with Tukey's test was used for two independent variables. Differences were considered statistically significant at *P* < 0.05.

### Data availability

RNA-seq data from this study is available in the NCBI GEO database (accession code GSE232751, https://www.ncbi.nlm.nih.gov/geo/query/acc.cgi?acc=GSE232751, reviewer token mjkluckqdtchxcp).

## Results

### Tet1 is significantly upregulated in AKI and AKI to CKD stages after I/R injury

**Figure 1:**
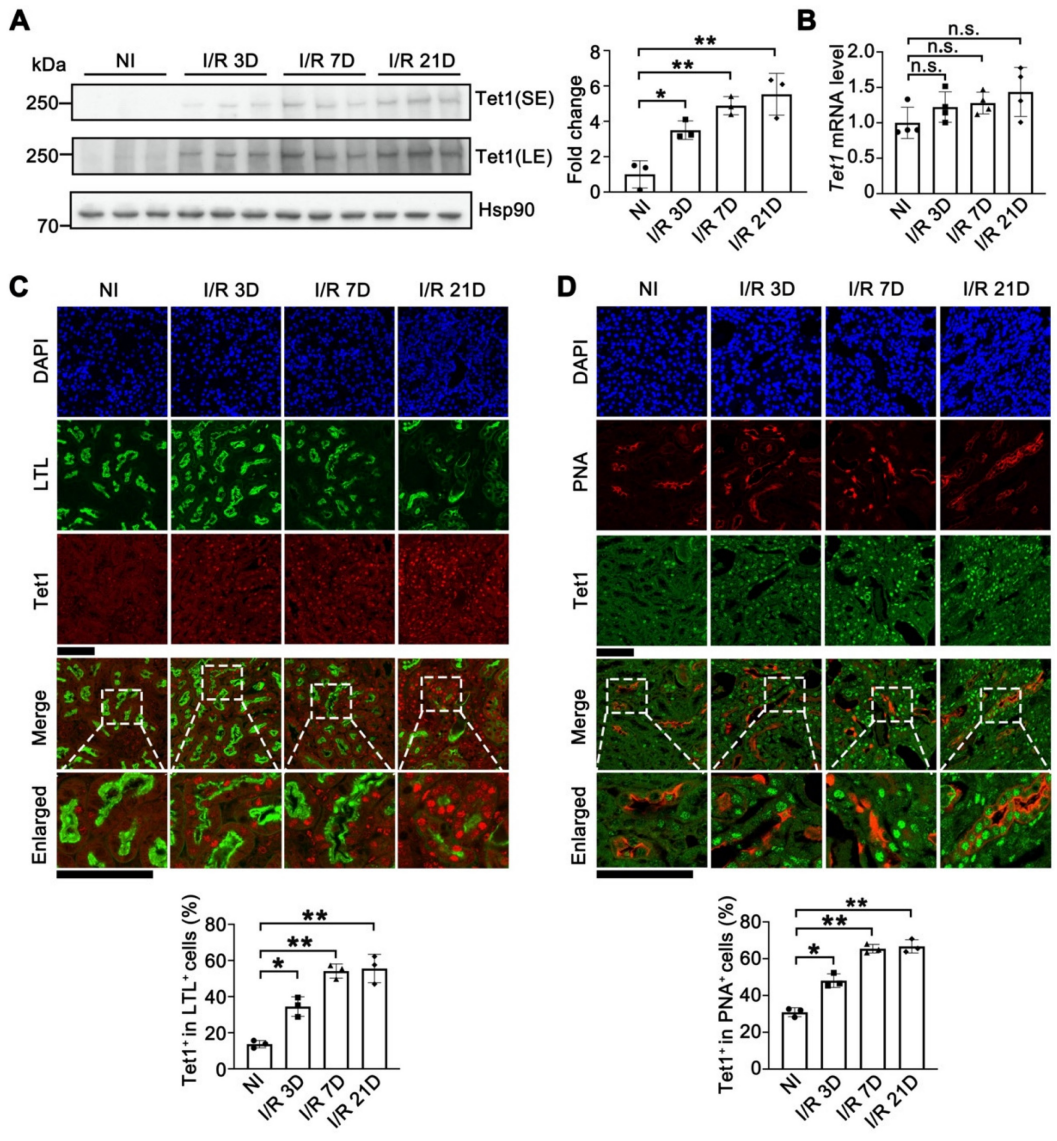
** Tet1 senses renal I/R injury. (A)** Western blot of Tet1 (left) with quantitative results (right) of non-injured (NI) or I/R injured mice at 3, 7, or 21 days after I/R injury (I/R 3D, I/R 7D, I/R 21D); SE, short exposure; LE, long exposure. **(B)** mRNA level of *Tet1* in the kidney at indicated times after I/R injury. **(C)** Representative co-immunofluorescent staining for Tet1 (red) with LTL (lotus tetragonolobus lectin; green) in the kidney, and quantitative results of the percentage of Tet1^+^ cells in LTL^+^ tubular cells. DAPI (blue) stains nuclei; scale bars = 50 μm. **(D)** Representative co-immunofluorescent staining for Tet1 (green) with PNA (peanut agglutinin; red) in the kidney, and quantitative results of the percentage of Tet1^+^ cells in PNA^+^ tubular cells. DAPI (blue) stains nuclei; scale bars = 50 μm. *P < 0.05; **P < 0.01; n.s., not significant.

To investigate the role of Tet1 in I/R-induced renal injury, the protein level of Tet1 was determined in mice at different time points following injury. Although the Tet1 level was relatively low in non-injured kidneys, Tet1 was significantly increased at day 3 and day 7 (AKI stage, I/R 3D and I/R7D), and remained high at day 21 (AKI to CKD stage, I/R 21D) after I/R injury in female mice (Figure 1A). Immunohistochemical studies consistently demonstrated weak Tet1 staining in renal glomeruli and tubular cells under non-injury conditions; after I/R injury, a significant increase in the nuclear Tet1 level was found in renal tubules and glomeruli at the AKI stage and the AKI to CKD stage with no gender-based differences (Figure S1A-B). However, no significant change in the transcriptional level of *Tet1* was observed in mouse kidney upon renal I/R injury (Figure 1B and Figure S1C). Post-translational modifications affect protein stability, structure and function 37. Ubiquitination or PARylation of Tet1 have been shown to alter its stability 38, 39. Co-immunoprecipitation assays were performed to determine whether Tet1 was PARylated or ubiquitinated following I/R injury. PARylation, but not ubiquitination, of Tet1 was increased in the kidneys at I/R 3D, suggesting increased PARylation of Tet1 may contribute to accumulation of renal Tet1 protein level after I/R injury (Figure S1D).

The kidney consists of multiple cell types with different functions. To identify the type of renal tubular cells that harbor increased Tet1, we co-stained cells for Tet1 and with different renal tubular markers, including LTL (lotus tetragonolobus lectin, detecting proximal tubules), PNA (peanut agglutinin, detecting distal tubules and collecting ducts), and DBA (dolichos biflorus agglutinin, detecting collecting ducts). While weak Tet1 staining was found in the proximal tubular cells, distal tubular cells, and collecting tubular cells under non-injury conditions, significantly increased nuclear Tet1 staining was found in these tubular cells at the AKI stage and the AKI to CKD stage in injured female mice (Figure 1C-D and Figure S1E). Similar results were found in male mice (data not shown). Interestingly, increased Tet1 protein level was found in TCMK1 cells upon TGF-β treatment, indicating that Tet1 protein may response to fibrotic stimulation *per se* (Figure S2). The persistent upregulation of Tet1 suggested its potential role in the pathogenesis of both AKI and the following AKI to CKD transition.

### Knockout of *Tet1* exacerbates I/R injury at both AKI stage and the AKI to CKD stage

To study the role of Tet1 in renal I/R injury, *Tet1* knockout (*Tet1* KO;* Tet1-/-*) mice and their gender-, age-matched wildtype (WT; *Tet1*^+/+^) littermates were studied (Figure S3A). The deletion efficiency of Tet1 in the kidney was examined. *Tet1* mRNA was barely detectable, while *Tet2* and *Tet3* mRNA levels were unaffected in the kidneys of *Tet1* KO mice (Figure S3B). Immunochemical staining further showed a significant reduction in the Tet1 protein level in the cortex and medulla of *Tet1* KO female mice (Figure S3C). Similar results were observed in male mice (data not shown). We and others have reported that *Tet1* KO mice are born at approximately 30% of the expected Mendelian ratio with a slight reduction in body size, which may be due to defects in embryonic development, while the *Tet1* KO mice that survive are viable 17, 40. At 12 weeks of age, adult* Tet1* KO mice showed similar renal morphology, number of glomeruli and kidney weight compared with those of their WT littermates (Figure S3D-F). Furthermore, normal renal function, as indicated by serum creatinine and BUN (blood urea nitrogen) levels, was found in *Tet1* KO female mice (Figure S3G). Similar results were found in adult male mice (data not shown).

**Figure 2:**
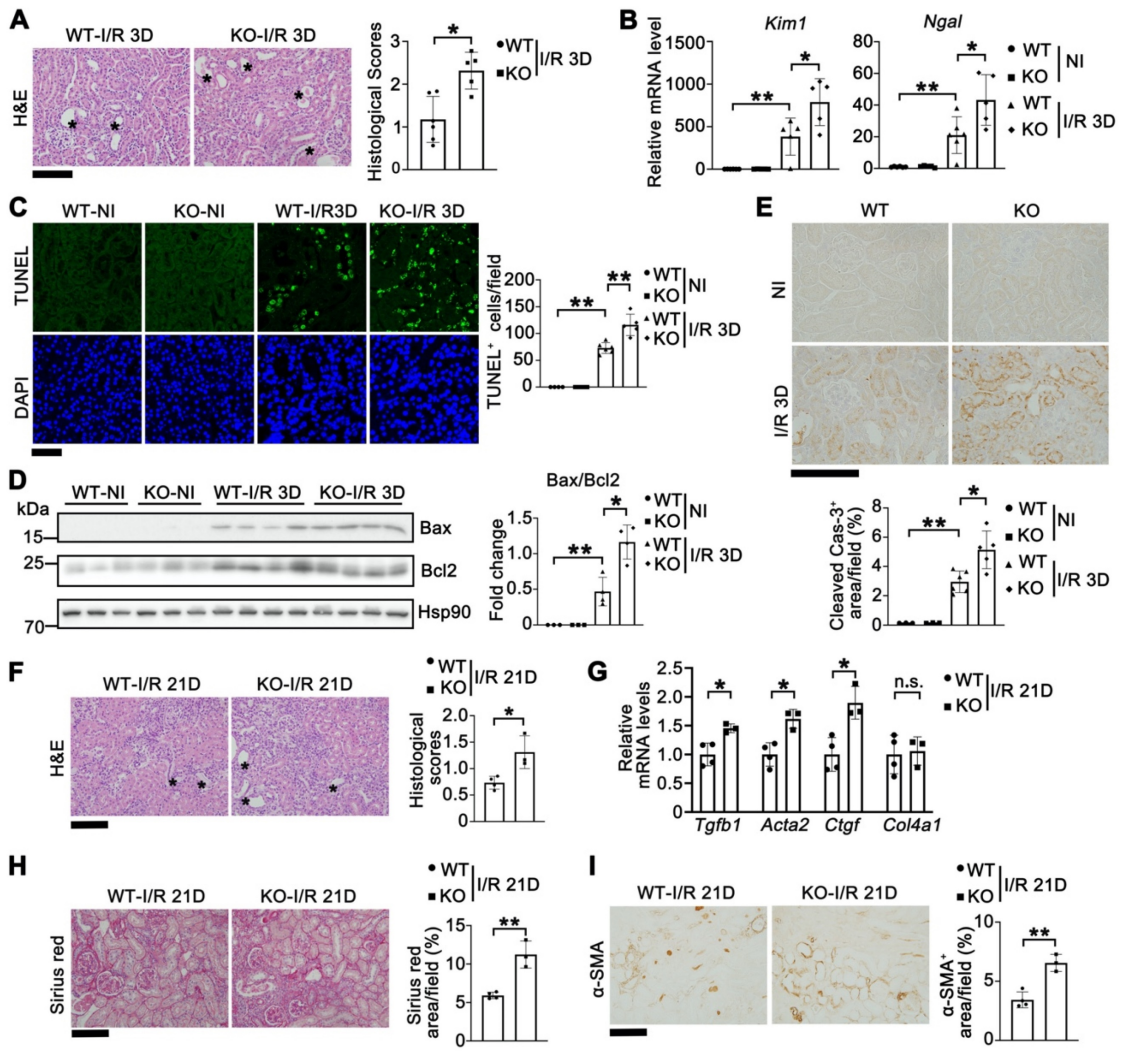
** Tet1 knockout increases I/R-induced acute kidney injury and accelerates the progression of AKI to CKD in female mice. (A)** Representative H&E images (left) with injury scores (right) of the kidney of WT and *Tet1* KO mice at 3 days (I/R 3D) after renal I/R injury. Asterisks indicate injured tubules. Scale bar = 100 μm. **(B)** mRNA levels of *Kim1* and *Ngal* in the kidney of WT and *Tet1* KO mice with or without injury. **(C)** Representative TUNEL images (left) with quantitative results (right) of WT and *Tet1* KO mice with or without injury. DAPI stains nuclei. Scale bar = 50 μm. **(D)** Western blots of Bax and Bcl2 (left) with quantitative results (right) in the kidney of WT and *Tet1* KO mice with or without injury. **(E)** Representative immunohistochemical staining of cleaved Caspase-3 in the kidney of WT and *Tet1* KO mice with or without injury. Scale bar = 100 μm. **(F)** Representative images of H&E staining of the kidney of WT and *Tet1* KO mice at 21 days (I/R 21D) after the renal I/R injury. Scale bar = 100 μm. **(G)** qPCR analysis of indicated genes in the kidney of WT and *Tet1* KO mice at I/R 21D. **(H-I)** Representative images of Sirius Red staining (H) and immunostaining for α-SMA (I) of the kidney of WT and *Tet1* KO mice at I/R 21D, and quantitative results of injury score. Scale bars = 100 μm. *P < 0.05; **P < 0.01.

Since Tet1 was elevated in both AKI and AKI to CDK stages (Figure 1), we first studied its role in the AKI stage (I/R 3D) in both male and female mice. *Tet1* KO female mice exhibited more severe renal morphological injury than WT mice at I/R 3D (Figure 2A). Significantly higher transcriptional levels of *Kim1* (kidney injury molecule 1) and *Ngal* (neutrophil gelatinase associated lipocalin) were consistently observed, indicating increased tubular damage in *Tet1* KO female mice at I/R 3D (Figure 2B). To explore whether the more severe renal injury observed in *Tet1* KO mice was due to increased cell death, a terminal deoxynucleotidyl transferase TUNEL (dUTP nick-end labeling) assay was performed. While there was no difference in the number of renal TUNEL^+^ cells between non-injured WT and *Tet1* KO female mice, an increase in cell death was observed in *Tet1* KO female mice compared to WT mice at I/R 3D (Figure 2C). Meanwhile, Western blots demonstrated an elevated Bax/Bcl2 ratio as well as an increase in cleaved Caspase-3 in the kidney of *Tet1* KO female mice at I/R 3D (Figure 2D-E). Similarly, *Tet1* KO male mice showed more severe pathological damage and cell death at I/R 3D (Figure S4), indicating that Tet1 deficiency increases renal I/R induced AKI in both genders.

To further assess the role of Tet1 in AKI to CKD transition, we collected renal samples at 21 days after renal I/R (I/R 21D). H&E staining revealed that *Tet1* KO female mice had more severe damage than WT female mice at I/R 21D (Figure 2F). qPCR results demonstrated that fibrotic related genes such as *Tgfb1* (transforming growth factor beta 1), *Acta2* (actin alpha 2) and *Ctgf* (connective tissue growth factor) were significantly upregulated in kidneys of *Tet1* KO mice compared with WT mice at I/R 21D (Figure 2G). Sirius red staining and immunohistochemical staining for α-SMA all demonstrated increased renal fibrosis in WT mice, which was further increased in *Tet1* KO female mice at I/R 21D (Figure 2H-I). In contrast, there was a similar level of pathological injury in injured WT and *Tet1* KO male mice at I/R 21D (Figure S5). Thus, losing Tet1 aggravated the AKI to CKD transition induced by renal I/R, at least in female mice. Based on this observation, the following experiments were carried out using female mice.

### Tet1 knockout reduces renal 5hmC and promotes inflammation in I/R injury

Recent studies suggest that Tet1 plays a role in a variety of diseases and biological processes by regulating DNA methylation and genome remodeling 41, 42. To establish whether depletion of Tet1 affects renal methylation/hydroxymethylation and gene transcription levels, we first examined the global levels of 5mC and 5hmC and analyzed the gene expression profile in the kidney at I/R 3D. Immunohistochemical and dot blot results showed that the level of renal 5hmC was significantly decreased in injured WT mice, which was further decreased in injured *Tet1* KO mice at I/R 3D; while there was no significant difference between non-injured WT and *Tet1* KO female mice (Figure S6A-B). However, there was no significant difference in the renal 5mC level between WT and *Tet1* KO female mice under non-injury and I/R 3D injured conditions (Figure S6B).

**Figure 3:**
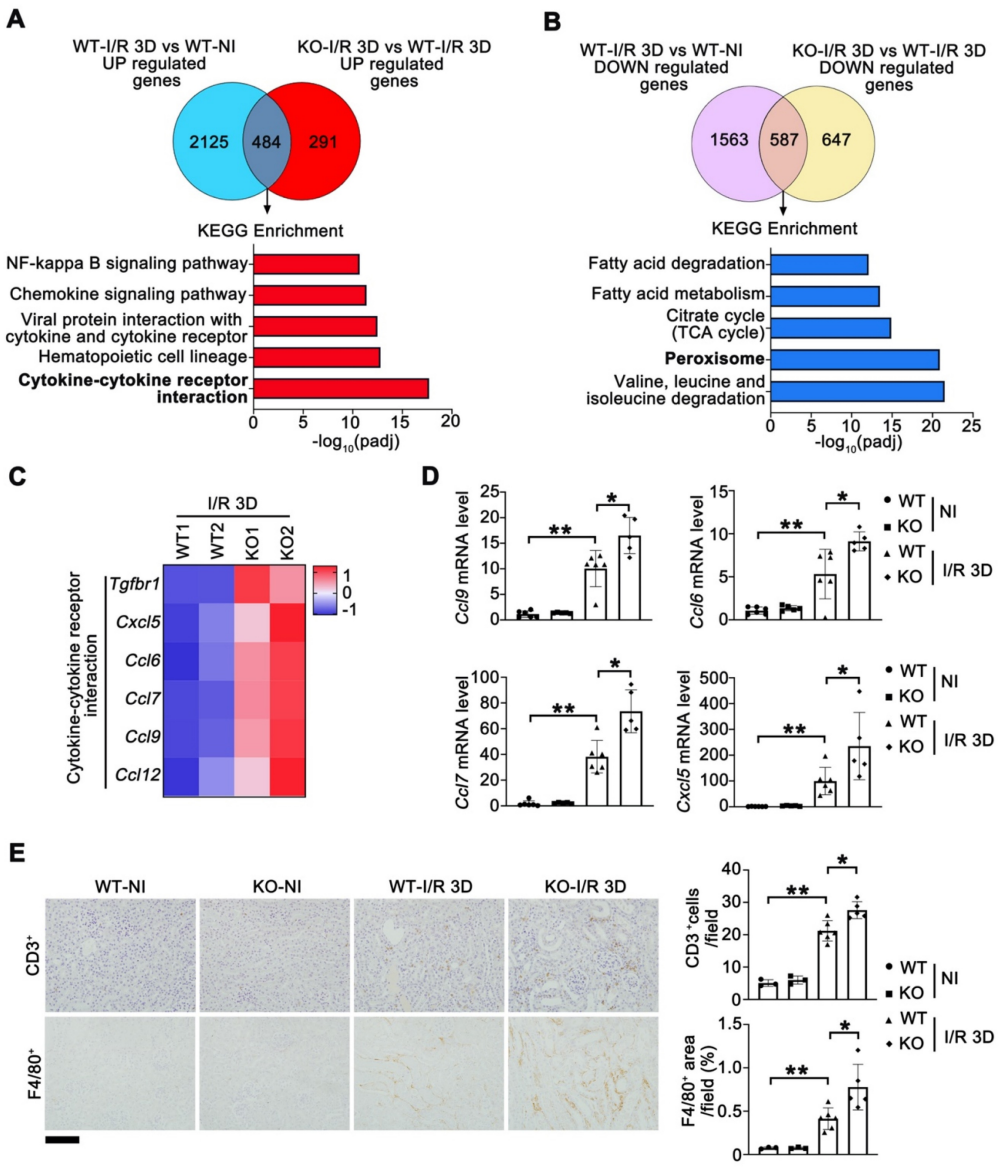
** RNA-sequencing analysis reveals that Tet1 knockout affects peroxisome and cytokine-cytokine receptor interaction in I/R injured female mice. (A-B)** KEGG pathways of co-upregulated and co-downregulated genes in KO-I/R 3D* vs*. WT-I/R 3D and WT-I/R 3D *vs*. WT-NI. **(C)** Heat map of inflammation-related pathways identified by RNA-seq. **(D)** qPCR analysis of indicated genes in the kidney of WT and *Tet1*KO mice with or without injury. **(E)** Representative immunostaining for CD3 (top) and F4/80 (bottom) with quantitative results (right) of the kidney of WT and *Tet1* KO mice at I/R 3D. Scale bar = 100 μm. Brown color indicates positive staining; *P < 0.05; **P < 0.01.

To determine the genes exhibiting altered expression in injured *Tet1* KO and WT female mice at I/R 3D, we analyzed the global transcriptome. Compared to non-injured kidneys, 2609/2150 significantly upregulated/downregulated genes were found at I/R 3D in injured kidneys of WT mice (Figure S7A). Meanwhile, *Tet1* knockout resulted in significant upregulation of 775 genes and downregulation of 1234 genes compared with those of WT mice at I/R 3D (Figure S7B). Since *Tet1* knockout exacerbated renal injury, we focused on the 484 genes that were further upregulated and the 587 genes that were further downregulated in injured *Tet1* KO mice (Figure 3A-B). At I/R 3D, KEGG enrichment showed that some of I/R-upregulated immune and inflammatory pathways, such as the cytokine-cytokine receptor interaction pathway, were further upregulated by *Tet1* ablation; while I/R-downregulated peroxisome as well as valine, leucine and isoleucine degradation pathways were further downregulated by *Tet1* ablation (Figure 3A-B).

Altered expression of genes involved in cytokine-cytokine receptor interaction was verified by qPCR (Figure 3C-D). Transcription of *Ccl6* (C-C motif chemokine ligand 6)*, Ccl7, Ccl9* and *Cxcl5* (C-X-C motif chemokine ligand 15) was increased in the kidneys of injured WT mice and was further upregulated in injured* Tet1* KO mice at I/R 3D (Figure 3D). Excessive expression of cytokines in the kidneys of KO mice may increase the infiltration of inflammatory cells and further aggravate tissue inflammation. As expected, the number of infiltrating macrophages (F4/80^+^ cells) and lymphocytes (CD3^+^ cells) was increased in the injured kidneys of WT mice, and was further increased in the injured kidneys of *Tet1* KO mice at I/R 3D (Figure 3E).

### Tet1 knockout increases oxidative stress in renal I/R injury

**Figure 4:**
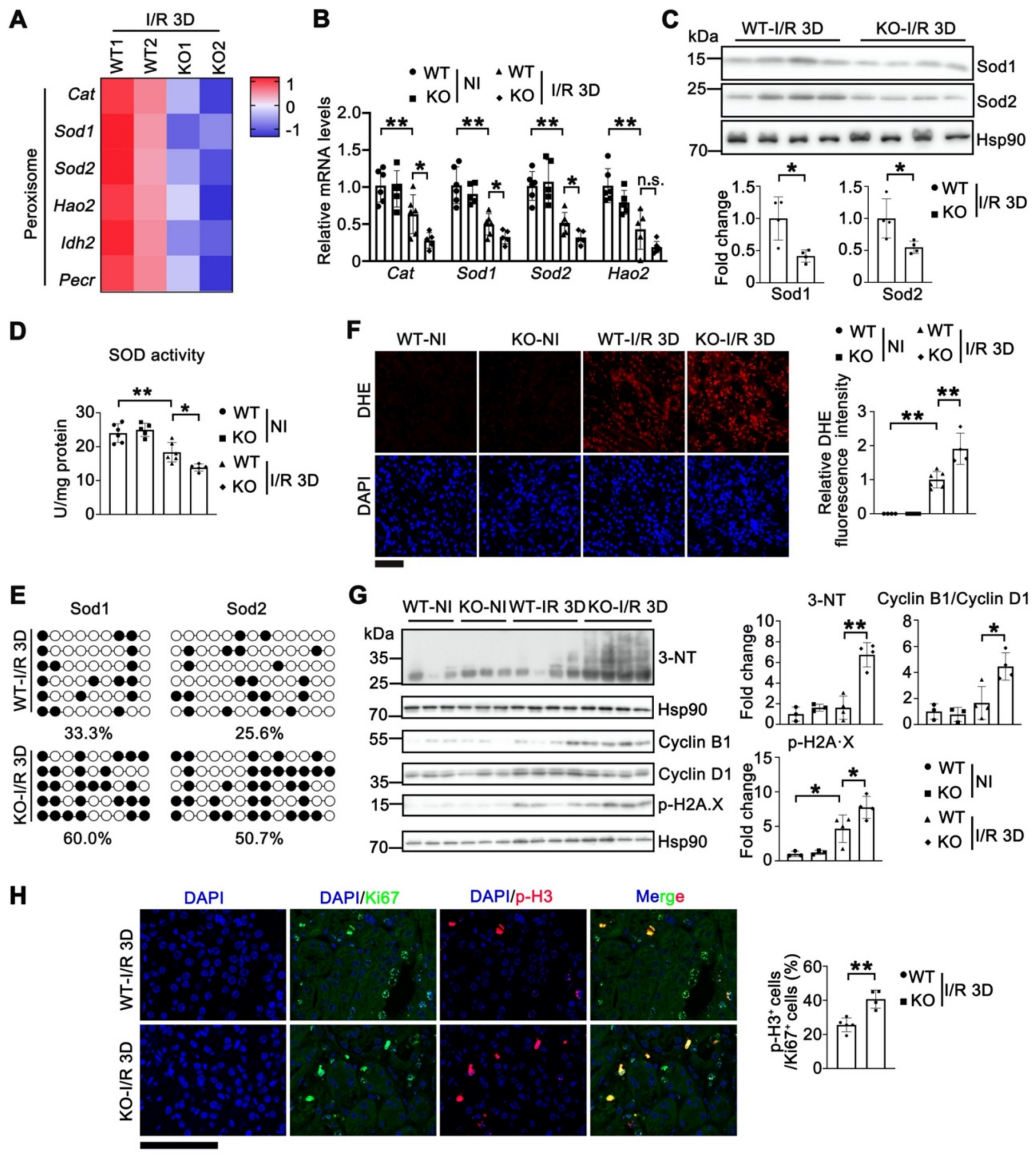
** Knockout of Tet1 increases I/R-induced oxidative stress. (A)** Heat map of peroxisome pathway identified by RNA-seq. **(B)** qPCR validations of indicated genes related to peroxisome pathway in the kidney of WT and *Tet1* KO mice with or without injury. **(C)** Western blots of Sod1 and Sod2 (top) with quantitative results (bottom) in the kidney of WT and *Tet1* KO mice at I/R 3D. **(D)** Total SOD activity in the kidney of WT and *Tet1* KO mice with or without injury. **(E)** Methylation status of the Sod1 and Sod2 promoters in the kidney of WT and *Tet1* KO mice at renal I/R 3D analyzed by bisulfite sequencing. Each row of dots represents CpG sites in a single sample, with each white dot indicating a single unmethylated CpG and each black dot indicating a single methylated CpG. **(F)** Representative DHE staining images with quantitative result in the kidney of WT and *Tet1* KO mice with or without injury. DAPI stains nuclei. Scale bar = 100 μm. **(G)** Western blot of 3-NT, Cyclin B1, Cyclin D1 and p-H2A·X with quantitative results in the kidney of WT and *Tet1* KO mice with or without injury. **(H)** Representative immunostaining for Ki67 and p-H3 with quantitative results of the kidney of WT and *Tet1* KO mice at I/R 3D. Scale bar = 50 μm. *P < 0.05; **P < 0.01; n.s., not significant.

Analysis of RNA sequencing (RNA-seq) data in the kidneys of *Tet1* KO female mice suggested that the number of downregulated genes was nearly twice that of upregulated genes, together with a reduction in 5hmC at I/R 3D (Figure 3A-B and Figure S6), which indicated that Tet1 may be more prone to activating than inhibiting gene expression in the kidney. The further downregulated peroxisome pathway in injured *Tet1* KO female mice caught our attention (Figure 3B), since the kidney is rich in peroxisomes containing antioxidant enzymes, particularly superoxide dismutase (SODs) and catalase (Cat), to regulate cellular redox homeostasis; while disrupted redox homeostasis is an important pathogenic driver in AKI 43, 44. Differences in the peroxisome antioxidant defense system associated genes between *Tet1* KO and WT mice identified by RNA-seq (Figure 4A) were further validated using qPCR. The results indicated that transcription of *Sod1*, *Sod2* and *Cat* were significantly decreased in WT kidneys after I/R injury, and was further downregulated in injured *Tet1* KO mice at the AKI stage (Figure 4B). Furthermore, decreased Sod1 and Sod2 protein levels were found in the kidneys of injured *Tet1* KO mice compared with those of injured WT mice at the AKI stage (Figure 4C). Meanwhile, total SOD activity was significantly decreased in WT kidneys, which was further decreased in injured kidneys of *Tet1* KO mice, after I/R 3D injury (Figure 4D). We next examined whether the altered transcription of *Sods* was due to a change in their methylation status. Bisulfite sequencing demonstrated increased DNA methylation on the CpG islands of *Sod1* and *Sod2* promoters in injured *Tet1* KO mice (Figure 4E).

Thus, we proposed that downregulated *Sods* and the decrease in their enzymatic activities may lead to accumulation of excessive reactive oxygen species (ROS), such as superoxide anions and hydroxyl radicals, as well as increased nitric oxide (NO)-derived peroxynitrite after the injury. Dihydroethidium (DHE) staining consistently demonstrated significantly increased ROS production in injured kidneys of WT mice, which was further increased in injured *Tet1* KO mice after I/R 3D injury (Figure 4F); consistently increased DHE staining was also found in* Tet1* KO mice at I/R 21D (Figure S8). Furthermore, 3-nitrotyrosine (3-NT) modified protein levels were increased in the injured kidneys of *Tet1* KO mice at the AKI stage, indicating increased peroxynitrite levels 45 (Figure 4G). ROS overload contributes to oxidative stress and DNA damage, which arrests the cell cycle at the G2/M phase by inhibiting the repair of damaged tubules, and leading to the acceleration of renal injury 9, 46. An increased in p-H2A.X and the cyclin B1/cyclin D1 ratio were consistently found in injured kidneys of *Tet1* KO mice at AKI stage, indicating more DNA damage and cell cycle arrest (Figure 4G). Furthermore, more accumulation of cells in the G2/M phase, as demonstrated by the increased p-H3/Ki67 ratio, was seen in injured kidneys of* Tet1* KO mice at the AKI stage (Figure 4H).

### Tet1 demethylase activity enhances the expression of Sod1 and Sod2

**Figure 5:**
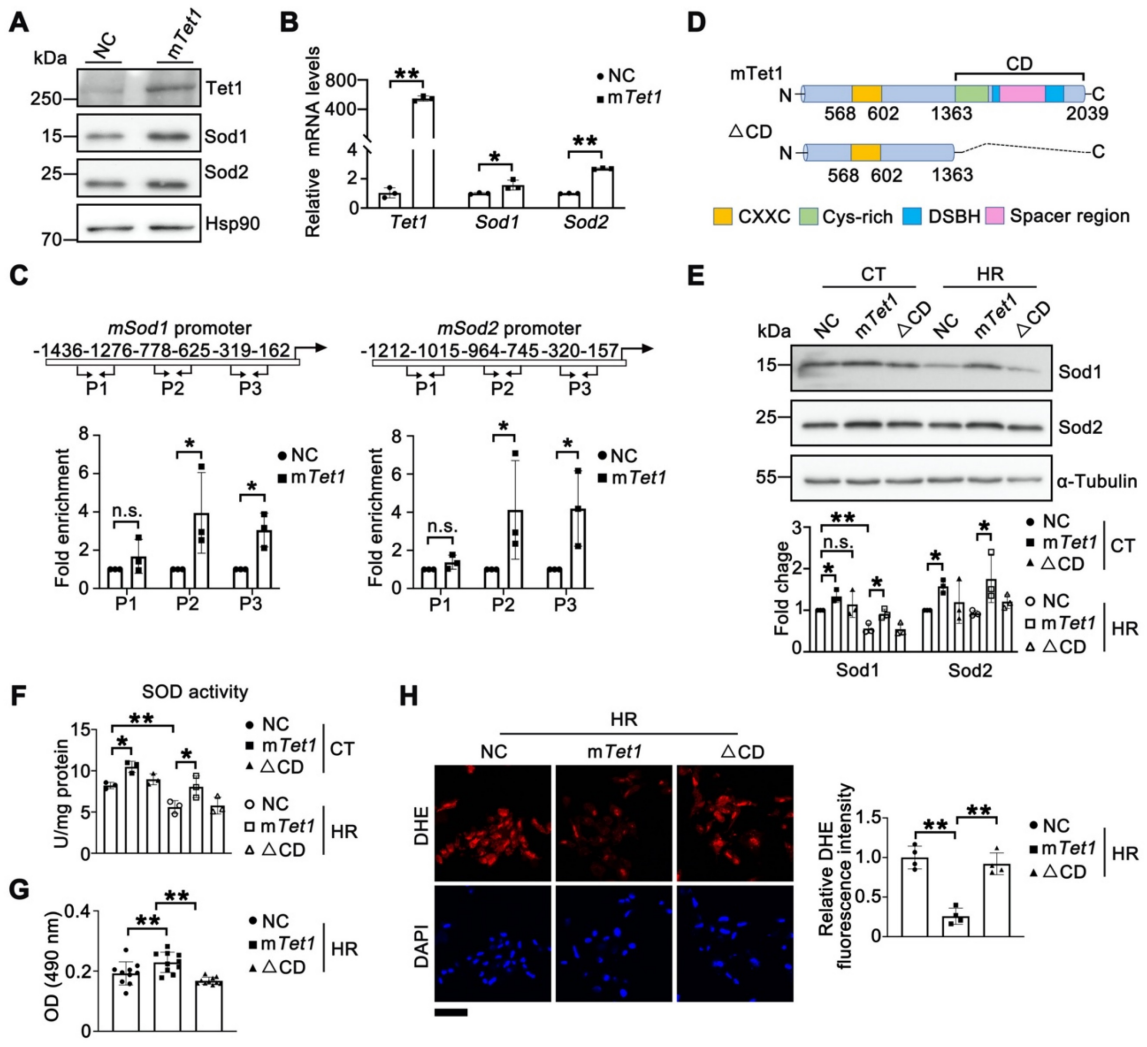
** Tet1 enzymatic activity enhances the expression of Sod1 and Sod2.** (**A**)Western blots of Sod1 and Sod2 in TCMK1 cells overexpressing full-length mouse *Tet1* (m*Tet1*) under normoxia. (**B**) mRNA levels of *Sod1* and *Sod2* in TCMK1 cells transfected with m*Tet1* under normoxia. (**C**) ChIP assay for Tet1 on the promoters of *Sod1* and *Sod2* in TCMK1 cells. Three different promoter regions of each gene were shown. (**D**) Schematic of the Tet1 domains. CD, Catalytic domain; CXXC, DNA-binding domain; Cys-rich, cysteine-rich domain; DSBH, double-stranded β-helix domain; Spacer region, Low complexity insert; (**E**) Western blots of Sod1 and Sod2 with quantitative results in TCMK1 cells transfected with m*Tet1* and its enzymatically inactive form (△CD) under normoxia and hypoxia for 12 h and reperfusion for 1 h (HR). (**F**) Total SOD activity in TCMK1 cells transfected with m*Tet1* and △CD under normoxia and HR. (**G**) MTT assay for TCMK1 cells overexpressing indicated Tet1 constructs after HR injury. (**H**) Representative DHE staining with quantitative results in TCMK1 cells overexpressing indicated Tet1 constructs under HR. The experiments were repeated three times, and at least three biological replicates per group were used. *P < 0.05; **P < 0.01; n.s., not significant.

Next, we explored whether Tet1 directly regulates Sod1 and Sod2. Western blots and qPCR analysis demonstrated that over-expression of full-length mouse *Tet1* (m*Tet1*) in TCMK1 cells significantly increased the mRNA and protein levels of Sod1 and Sod2 (Figure 5A-B). ChIP assays demonstrated direct binding of Tet1 to the promoter regions of *Sod1* and *Sod2* (Figure 5C). To investigate whether the regulation of *Sods* by Tet1 depend on its enzyme activity, a truncated form that abolishes enzymatic activity (m*Tet1*△CD) was used (Figure 5D). As expected, the protein levels of sod1 and sod2 were significantly increased after m*Tet1* overexpression under either normoxia or HR conditions, but not in m*Tet1*△CD overexpressed group (Figure 5E). Furthermore, total SOD activity was significantly increased in the m*Tet1* overexpression group, but not in the m*Tet1*△CD overexpression group under either normoxia or HR conditions (Figure 5F). MTT assay and DHE staining results also showed that overexpression of m*Tet1*, but not m*Tet1*△CD, could reduce ROS accumulation and enhance cell viability under HR conditions (Figure 5G-H). Taken together, these results suggest that Tet1 enhances Sod1 and Sod2 levels dependent on its demethylase activity.

### Tempol administration rescues I/R injury in *Tet1* KO mice

**Figure 6:**
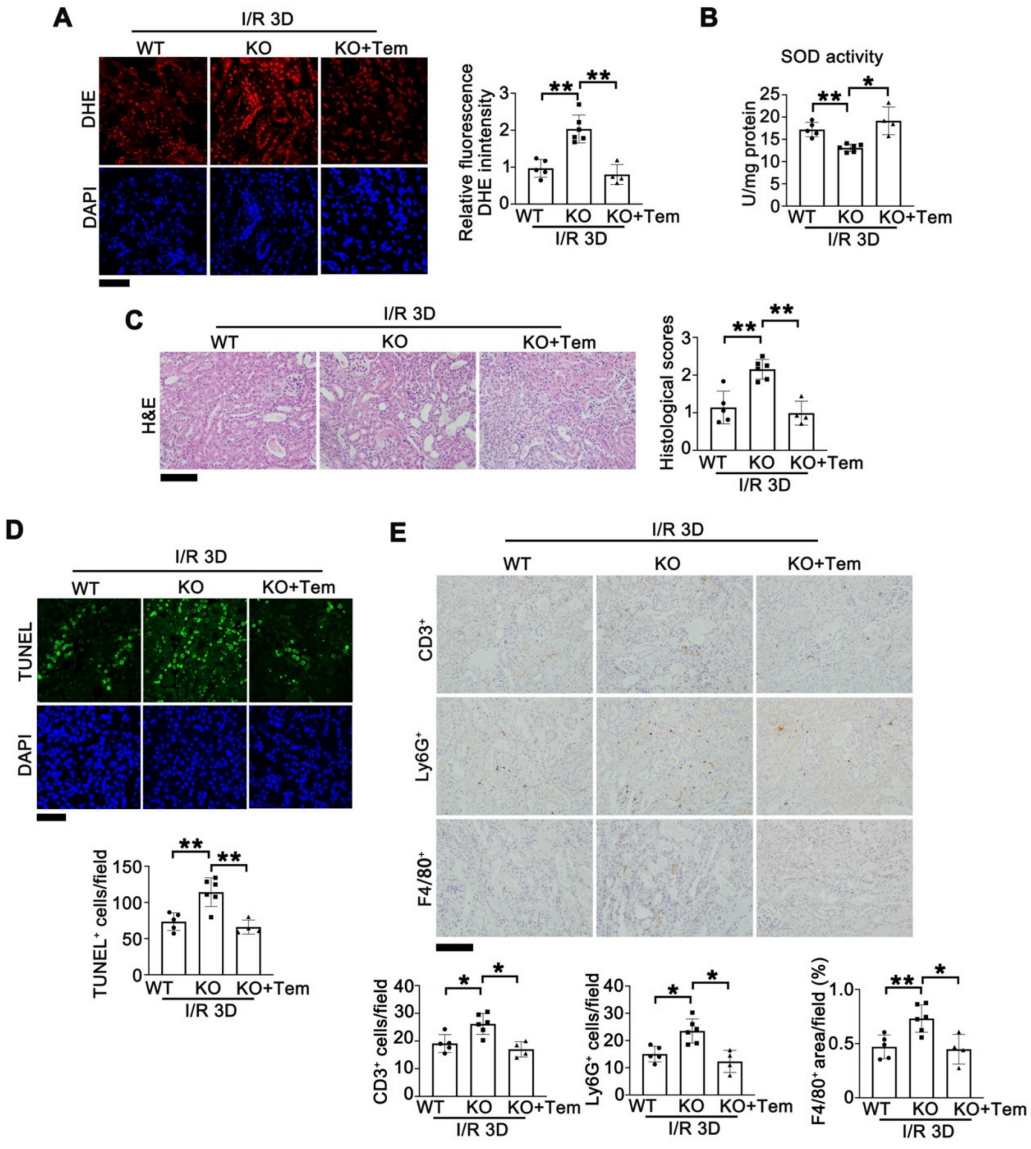
** Tempol administration rescues I/R-induced kidney injury in female *Tet1* KO mice. (A)** Representative DHE staining images (left) with quantitative results (right) in the kidney of indicated groups at I/R 3D. Scale bar = 50 μm. **(B)** Total SOD activity in the kidney of indicated groups at I/R 3D. **(C)** Representative H&E images (left) with injury scores (right) of the kidney of indicated groups at I/R 3D. Scale bar = 100 μm.** (D)** Representative TUNEL images (top) with quantitative results (bottom) in the kidneys of indicated groups at I/R 3D. Scale bar = 50 μm.** (E)** Representative immunohistochemical staining for CD3, Ly6G, F4/80 and quantitative results in the kidney of indicated groups at I/R 3D. Scale bar = 100 μm. *P < 0.05, **P < 0.01.

To investigate whether ROS overload caused by downregulated *Sods* are responsible for Tet1 deficiency enhanced I/R kidney injury, a membrane-permeable SOD mimetic, tempol, which is an FDA approved medication for cerebral cavernous hemangioma 47, was administered through oral gavage into *Tet1* KO mice after I/R injury. At I/R 3D, DHE staining demonstrated that the increased ROS in injured kidneys of *Tet1* KO mice was normalized by tempol (Figure 6A). The total SOD activity in injured kidneys of *Tet1* KO mice was also rescued by tempol treatment (Figure 6B). H&E staining showed that tempol alleviated the more severe renal injury in *Tet1* KO mice (Figure 6C). Similarly, TUNEL assay demonstrated that tempol completely abrogated the enhanced cell death in injured kidneys of* Tet1* KO mice (Figure 6D). Moreover, immunohistochemical results of F4/80^+^, CD3^+^ and Ly6G^+^ showed that the increased inflammatory cell infiltration in injured kidneys of *Tet1* KO mice was decreased after tempol treatment (Figure 6E). These results suggested that tempol-mediated ROS elimination rescued the exacerbated renal injury in *Tet1* KO mice.

### Knockout of Tet1 exacerbates UUO-induced renal injury

**Figure 7:**
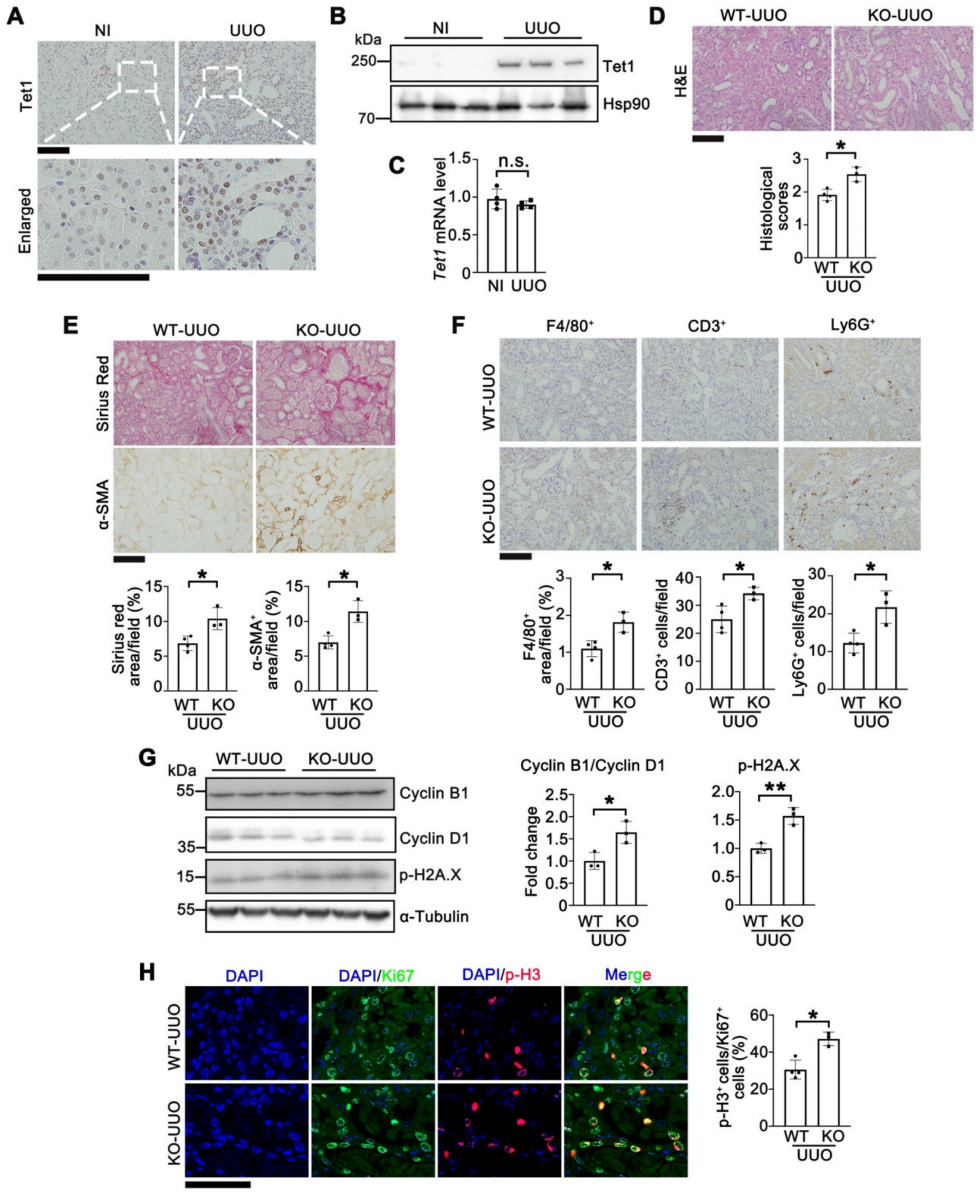
** Knockout of Tet1 in female mice increases UUO-induced kidney injury. (A)** Representative immunostaining of Tet1 in the kidney of WT mice with or without UUO injury. Scale bar = 100 μm. **(B-C)** Western blot **(B)** and qPCR **(C)** analysis of *Tet1* in the kidney of WT mice with or without UUO injury. **(D)** Representative H&E images with injury scores of the kidney of WT and *Tet1* KO mice at UUO 7D. Scale bar = 100 μm. **(E)** Representative images of Sirius Red staining and immunostaining for α-SMA with quantitative results. Scale bar = 100 μm. **(F)** Representative immunostaining for F4/80, CD3, Ly6G and quantitative results. Scale bar = 100 μm. **(G)** Western blot of cyclin B1, cyclin D1 and p-H2A·X with quantitative results in the kidney of WT and *Tet1* KO mice at UUO 7D. **(H)** Representative immunostaining for Ki67, p-H3 and quantitative results. Scale bar = 50 μm. *P < 0.05. **P < 0.01; n.s., not significant.

To investigate whether Tet1 responds to other renal injury models, we examined the expression of Tet1 in UUO- or folic acid-induced renal injury models. Like I/R-induced renal injury, Tet1 was significantly increased at day 7 after UUO injury or at day 7 after folic acid overdose injury, while its mRNA level was unchanged (Figure 7A-C and Figure S9). To exclude model-specific effects, the role of Tet1 in renal protection was further investigated in the UUO model. *Tet1* KO mice consistently exhibited more severe renal pathological injury than WT mice at day 7 after UUO injury (Figure 7D). Both α-SMA staining and Sirius red staining showed more severe fibrosis in *Tet1* KO female mice compared to WT mice after UUO injury (Figure 7E). Moreover, the number of infiltrating macrophage (F4/80^+^), T cell (CD3^+^) and neutrophil (Ly6G^+^) was increased in *Tet1* KO mice after UUO injury (Figure 7F); while immunofluorescent staining for p-H3 and Ki67 as well as Western blots for Cyclin B1 and Cyclin D1 indicated more severe G2/M cell cycle arrest in the kidneys of *Tet1* KO mice after UUO injury (Figure 7G-H). Increased DNA damage, as demonstrated by p-H2A.X level, was also observed in the kidneys of *Tet1* KO female mice after UUO injury (Figure 7G). These results demonstrated that knockout of Tet1 exacerbated UUO induced renal injury.

### Knockout of Tet1 increases UUO-induced oxidative stress

**Figure 8:**
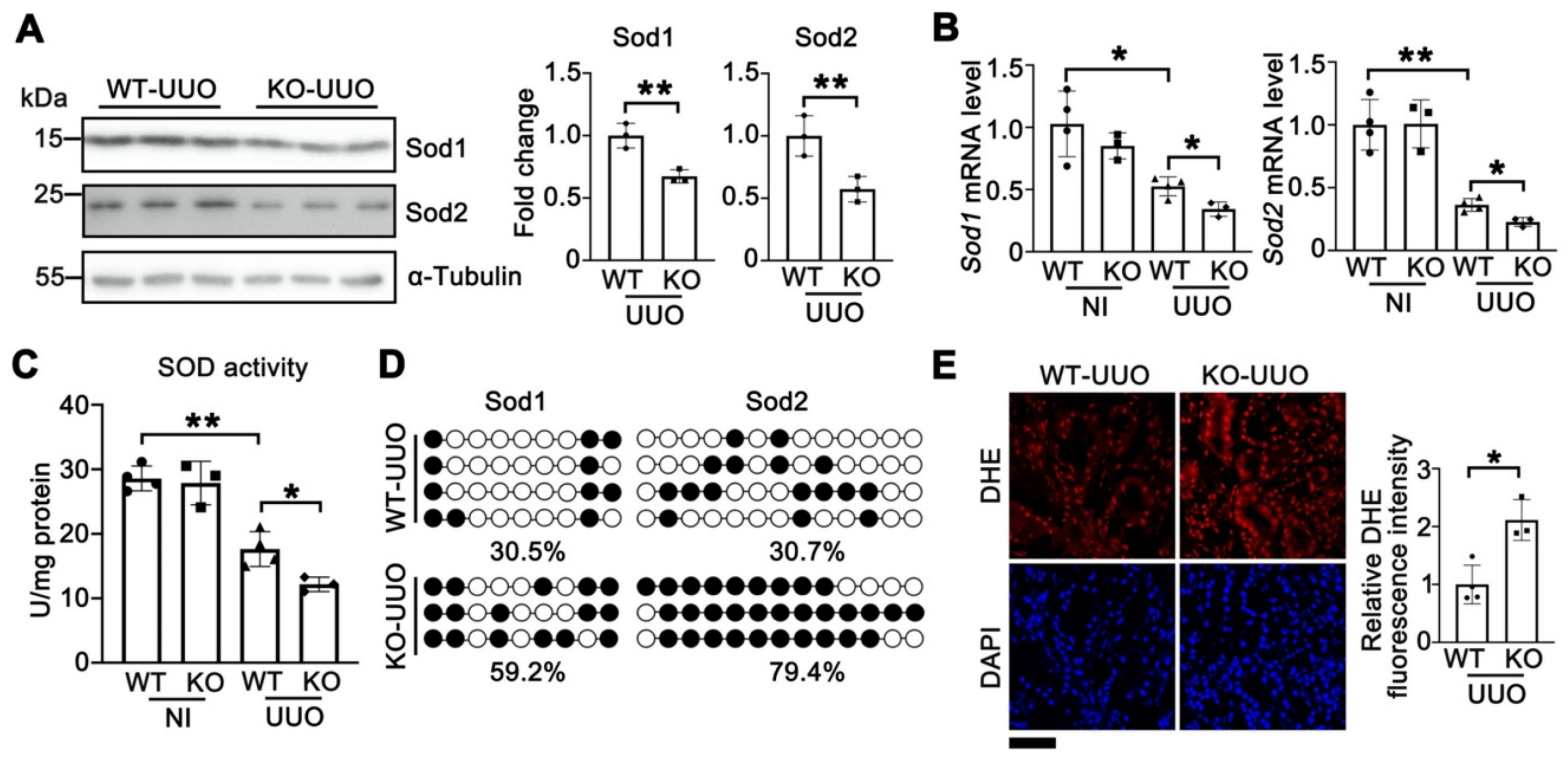
**Knockout of Tet1 increases UUO-induced oxidative stress. (A)** Western blots of Sod1, Sod2 with quantitative results in the kidney of WT and *Tet1* KO mice at UUO 7D. **(B)** mRNA levels of Sod1 and Sod2 in the kidney of WT and *Tet1* KO mice with or without UUO injury. **(C)** Total SOD activity in the kidney of WT and *Tet1* KO mice with or without UUO injury. **(D)** Methylation status of *Sod1* and *Sod2* promoters in the kidney of WT and *Tet1* KO mice at UUO 7D analyzed by bisulfite sequencing. Each row of dots represents CpG sites in a single sample, with each white dot indicates a single unmethylated CpG and each black dot indicates a single methylated CpG. **(E)** Representative DHE staining images with quantitative result of WT and* Tet1* KO mice with or without UUO injury. DAPI stains nuclei. Scale bar = 50 μm. *P < 0.05. **P < 0.01.

To investigate whether the Tet1-Sods-ROS axis also existed in UUO-induced renal injury, the levels of Sod1 and Sod2 were examined. Upon UUO injury, the protein and mRNA levels of Sod1 and Sod2 were significantly reduced in *Tet1* KO mice compared with those of WT mice (Figure 8A-B). Furthermore, the total SOD activity was decreased in *Tet1* KO mice after UUO injury (Figure 8C). Bisulfite sequencing demonstrated that the DNA methylation levels in the promoters of *Sod1* and *Sod2* were increased in *Tet1* KO mice compared with those of WT mice (Figure 8D). In addition, DHE staining demonstrated increased ROS level in the kidneys of *Tet1* KO mice after UUO injury (Figure 8E). These results indicated that in UUO-mediated renal injury, knockout of Tet1 downregulated Sod1 and Sod2 expression and increased oxidative stress.

### Tempol administration rescues UUO-induced kidney injury in *Tet1* KO mice

**Figure 9:**
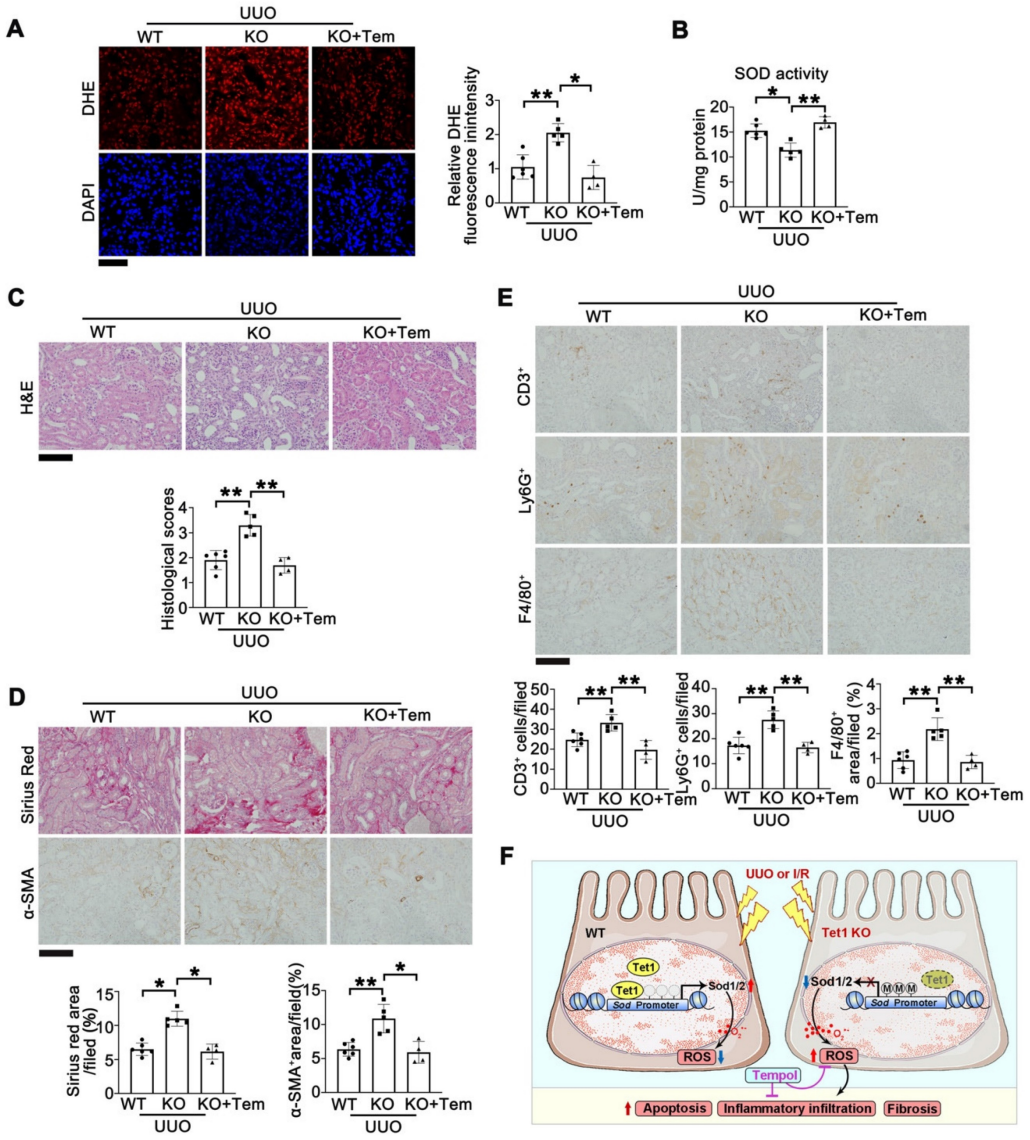
** Tempol administration rescues UUO-induced kidney injury in female *Tet1* KO mice. (A)** Representative DHE staining images (left) with quantitative results (right) in the kidney of WT and *Tet1* KO mice with or without tempol treatment at UUO 7D. Scale bar = 50 μm.** (B)** Total SOD activity in the kidney of WT and *Tet1* KO mice with or without tempol treatment at UUO 7D. **(C)** Representative H&E images with injury scores of the kidney of WT and *Tet1* KO mice with or without tempol treatment at UUO 7D. Scale bar = 100 μm. **(D)** Representative images of Sirius Red staining and immunostaining for α-SMA with quantitative results in the kidney of WT and *Tet1* KO mice with or without tempol treatment at UUO 7D. Scale bar = 100 μm. **(E)** Representative immunohistochemical staining for CD3, Ly6G, F4/80 and quantitative results in the kidney of WT and *Tet1* KO mice with or without tempol treatment at UUO 7D. Scale bar = 100 μm. **(F)** A schematic model for the role that Tet1 plays in I/R- or UUO-induced kidney injury. *P < 0.05; **P < 0.01.

Finally, we investigated whether tempol could also rescue UUO-induced renal injury in* Tet1* KO mice. Tempol was administered through oral gavage into *Tet1* KO mice after surgery. Seven days after UUO injury, DHE staining demonstrated that increased ROS in injured kidneys of *Tet1* KO mice was rescued by tempol (Figure 9A). The reduction in total SOD activity in injured kidneys of *Tet1* KO mice was also rescued by tempol treatment (Figure 9B). H&E staining showed that the more severe renal injury in *Tet1* KO mice was alleviated by tempol treatment (Figure 9C). Similarly, Sirius red and α-SMA staining demonstrated that tempol administration completely abrogated the enhanced fibrosis in injured* Tet1* KO mice (Figure 9D). Moreover, immunohistochemical staining for CD3^+^, Ly6G^+^and F4/80^+^ showed that the increased infiltration of inflammatory cells in injured kidneys of *Tet1* KO mice was decreased after tempol injection (Figure 9E). These results suggested that tempol treatment rescued the exacerbated kidney injury in *Tet1* KO mice following UUO.

## Discussion

Abnormal DNA methylation has been found in a variety of diseases. As important regulators for DNA methylation, Tet1 and its family members have received extensive attention for their DNA demethylation function 48. For example, Tet1 promotes fatty acid oxidation and inhibits NAFLD progression by hydroxymethylation of the *PPARα* (peroxisome proliferator-activated receptor alpha) promoter 49. *Tet1* deficiency reduces 5hmC levels in spermatogonia and downregulates a subset of genes critical for cell cycle, germ cell differentiation, and meiosis, which leads to premature reproductive senescence 50. For other Tet enzymes, studies on Tet2 KO mice suggested that Tet2 protects renal I/R injury by repressing inflammatory responses 51; while Tet3 mediates the hydroxymethylation of the* Rasal1* (RAS protein activator like 1) gene in renal fibrosis models 52, 53. Our study suggests that Tet1 plays a vital protective role in the acute and acute-to-chronic phases of renal injury.

Our current study demonstrated that Tet1 is significantly increased in multiple models of kidney injury while there was no significant change in *Tet1* mRNA levels in either model (Figures 1, 7, and S9). It has been reported that Tet1 interacts with poly (ADP-ribose) polymerase 1 (PARP1) and is targeted by covalent PARylation in its catalytic domain, which improves the stability of Tet1 and enhances its activity 38, 54. Notably, enhanced PARylation has been found in multiple renal diseases 55, 56. (VprBP-DDB1-CUL4-ROC1) E3 ubiquitin ligase-mediated ubiquitylation on Lys1537 of Tet1 affects its stability 39. Upon AKI injury, we found alteration of PARylation, but not ubiquitination, of Tet1 (Figure S1D). Thus, increased PARylation of Tet1 may contribute to increased Tet protein level during AKI, at least under I/R injury. However, the exact site(s) of Tet1 responsible for PARylation need to be determined. In addition, O-GlcNAc modification on Thr535 of Tet1 has also been reported to enhance its stability in mouse embryonic stem cells 57, however, whether this modification contributes to increased Tet1 protein level following renal injury needs further investigation.

Tet1 plays a protective role against oxidative stress in different tissues/cells. In our study, *Tet1* KO increased ROS accumulation in the kidney, in both the I/R or UUO models. Further study found that *Tet1* KO increased the methylation levels of the CpG island in the *Sod1/2* promoters, inhibited the expression of Sod1/2 and hindered the removal of superoxide (Figures 4 and 8). *In vitro,* Tet1 promotes the expression of Sod1/2 dependent on its enzymatic activity, and overexpression of m*Tet1* rescued oxidative stress induced by hypoxia (Figure 5). It has been reported that Tet1 upregulates Klotho expression through DNA demethylation, thereby inhibiting hydrogen peroxide-induced apoptosis in cerebellar granule cells 58. Moreover, Tet1 plays a protective role in arsenic induced oxidative stress in human bronchial epithelial cells by regulating the methylation of the *OGG1* (8-oxoguanine DNA glycosylase) and *GSTP1* (glutathione S-transferase Pi 1) promoters 41. All these results suggest that Tet1 plays a role in the response to oxidative stress through an enzyme-dependent mechanism, and this effect is not limited to specific tissues/cells.

Oxidative stress induces cell death, leading to inflammation, which in turn leads to kidney injury 59, 60. We consistently found that I/R-induced apoptosis and inflammation are dependent on oxidative stress. In our study, the levels of ROS, apoptotic proteins such as Bax/Bcl2, pro-inflammatory genes and immune cell markers in the kidneys of *Tet1* KO mice were higher than those of WT mice following I/R injury (Figures 2 and 3). After injection of tempol, a ROS scavenger, both apoptosis and inflammation were reduced in I/R injured *Tet1* KO mice along with decreased ROS (Figure 6). We observed the same phenomenon in the UUO model (Figure 9). Therefore, removal of excess reactive oxygen species by activating the antioxidant system may be a key to treating AKI.

In summary, we report increased Tet1 expression in the models of I/R- or UUO-induced AKI, AKI-CKD progression. More severe kidney injury was found in *Tet1* KO mice. Mechanistically, Tet1 promotes the expression of the key antioxidant enzyme Sod1/2 by reducing the methylation on their promoters, thus reducing the accumulation of ROS and the occurrence of inflammation, apoptosis and fibrosis in injured kidney (Figure 9F).

## Supplementary Material

Supplementary figures and tables.

## References

[1] Ronco C, Bellomo R, Kellum JA (2019). Acute kidney injury. Lancet.

[2] Vijayan A (2021). Tackling AKI: prevention, timing of dialysis and follow-up. Nat Rev Nephrol.

[3] Strausser SA, Nakano D, Souma T (2018). Acute kidney injury to chronic kidney disease transition: insufficient cellular stress response. Curr Opin Nephrol Hypertens.

[4] Zhao Z, Wu J, Xu H, Zhou C, Han B, Zhu H (2020). XJB-5-131 inhibited ferroptosis in tubular epithelial cells after ischemia-reperfusion injury. Cell Death Dis.

[5] Zhang J, Bi J, Ren Y, Du Z, Li T, Wang T (2021). Involvement of GPX4 in irisin's protection against ischemia reperfusion-induced acute kidney injury. J Cell Physiol.

[6] Zhao M, Wang Y, Li L, Liu S, Wang C, Yuan Y (2021). Mitochondrial ROS promote mitochondrial dysfunction and inflammation in ischemic acute kidney injury by disrupting TFAM-mediated mtDNA maintenance. Theranostics.

[7] Fu Y, Xiang Y, Li H, Chen A, Dong Z (2022). Inflammation in kidney repair: Mechanism and therapeutic potential. Pharmacol Ther.

[8] Bhargava P, Schnellmann RG (2017). Mitochondrial energetics in the kidney. Nat Rev Nephrol.

[9] Ferenbach DA, Bonventre JV (2015). Mechanisms of maladaptive repair after AKI leading to accelerated kidney ageing and CKD. Nat Rev Nephrol.

[10] Chevalier RL, Thornhill BA, Forbes MS, Kiley SC (2010). Mechanisms of renal injury and progression of renal disease in congenital obstructive nephropathy. Pediatr Nephrol.

[11] Ucero AC, Benito-Martin A, Izquierdo MC, Sanchez-Nino MD, Sanz AB, Ramos AM (2014). Unilateral ureteral obstruction: beyond obstruction. Int Urol Nephrol.

[12] Aranda-Rivera AK, Cruz-Gregorio A, Aparicio-Trejo OE, Ortega-Lozano AJ, Pedraza-Chaverri J (2021). Redox signaling pathways in unilateral ureteral obstruction (UUO)-induced renal fibrosis. Free Radic Biol Med.

[13] Sun W, Zang L, Shu Q, Li X (2014). From development to diseases: the role of 5hmC in brain. Genomics.

[14] Szulwach KE, Li X, Li Y, Song CX, Wu H, Dai Q (2011). 5-hmC-mediated epigenetic dynamics during postnatal neurodevelopment and aging. Nat Neurosci.

[15] Melamed P, Yosefzon Y, David C, Tsukerman A, Pnueli L (2018). Tet Enzymes, Variants, and Differential Effects on Function. Front Cell Dev Biol.

[16] Kohli RM, Zhang Y (2013). TET enzymes, TDG and the dynamics of DNA demethylation. Nature.

[17] Yuan Y, Liu C, Chen X, Sun Y, Xiong M, Fan Y (2021). Vitamin C Inhibits the Metabolic Changes Induced by Tet1 Insufficiency Under High Fat Diet Stress. Mol Nutr Food Res.

[18] Gu Y, Chen J, Zhang H, Shen Z, Liu H, Lv S (2020). Hydrogen sulfide attenuates renal fibrosis by inducing TET-dependent DNA demethylation on Klotho promoter. FASEB J.

[19] Wang J, Zhang D, Du J, Zhou C, Li Z, Liu X (2017). Tet1 facilitates hypoxia tolerance by stabilizing the HIF-alpha proteins independent of its methylcytosine dioxygenase activity. Nucleic Acids Res.

[20] Xiong M, Chen H, Fan Y, Jin M, Yang D, Chen Y (2023). Tubular Elabela-APJ axis attenuates ischemia-reperfusion induced acute kidney injury and the following AKI-CKD transition by protecting renal microcirculation. Theranostics.

[21] Bai Y, Wang W, Yin P, Gao J, Na L, Sun Y (2020). Ruxolitinib Alleviates Renal Interstitial Fibrosis in UUO Mice. Int J Biol Sci.

[22] Yuan Q, Lv Y, Ding H, Ke Q, Shi C, Luo J (2021). CPT1alpha maintains phenotype of tubules via mitochondrial respiration during kidney injury and repair. Cell Death Dis.

[23] Zhang G, Wang Q, Zhou Q, Wang R, Xu M, Wang H (2016). Protective Effect of Tempol on Acute Kidney Injury Through PI3K/Akt/Nrf2 Signaling Pathway. Kidney Blood Press Res.

[24] Ewees MG, Messiha BAS, Abdel-Bakky MS, Bayoumi AMA, Abo-Saif AA (2019). Tempol, a superoxide dismutase mimetic agent, reduces cisplatin-induced nephrotoxicity in rats. Drug Chem Toxicol.

[25] Chen H, Wang L, Wang WJ, Cheng C, Zhang Y, Zhou Y (2017). ELABELA and an ELABELA Fragment Protect against AKI. J Am Soc Nephrol.

[26] Wang Q, Chen Y, Xie Y, Yang D, Sun Y, Yuan Y (2022). Histone H1.2 promotes hepatocarcinogenesis by regulating signal transducer and activator of transcription 3 signaling. Cancer Sci.

[27] Wang J, Xiong MR, Fan Y, Liu CY, Wang Q, Yang D (2022). Mecp2 protects kidney from ischemia-reperfusion injury through transcriptional repressing IL-6/STAT3 signaling. Theranostics.

[28] Wang C, Xiong M, Yang C, Yang D, Zheng J, Fan Y (2020). PEGylated and Acylated Elabela Analogues Show Enhanced Receptor Binding, Prolonged Stability, and Remedy of Acute Kidney Injury. J Med Chem.

[29] Chen H, Liu C, Wang Q, Xiong MR, Zeng X, Yang D (2022). Renal UTX-PHGDH-serine axis regulates metabolic disorders in the kidney and liver. Nat Commun.

[30] Chen Y, Shi J, Wang X, Zhou L, Wang Q, Xie Y (2023). An antioxidant feedforward cycle coordinated by linker histone variant H1.2 and NRF2 that drives nonsmall cell lung cancer progression. Proc Natl Acad Sci U S A.

[31] Yang D, Fan Y, Xiong M, Chen Y, Zhou Y, Liu X Loss of renal tubular G9a benefits acute kidney injury by lowering focal lipid accumulation via CES1. EMBO Rep. 2023: e56128.

[32] Yang C, Xu H, Yang D, Xie Y, Xiong M, Fan Y (2023). A renal YY1-KIM1-DR5 axis regulates the progression of acute kidney injury. Nat Commun.

[33] Huang Y, Xie Y, Yang D, Xiong M, Chen X, Wu D (2022). Histone demethylase UTX aggravates acetaminophen overdose induced hepatotoxicity through dual mechanisms. Pharmacol Res.

[34] Zhang W, Yang D, Yuan Y, Liu C, Chen H, Zhang Y (2020). Muscular G9a Regulates Muscle-Liver-Fat Axis by Musclin Under Overnutrition in Female Mice. Diabetes.

[35] Yuan Y, Fan Y, Zhou Y, Qiu R, Kang W, Liu Y (2023). Linker histone variant H1.2 is a brake on white adipose tissue browning. Nat Commun.

[36] Sun Y, Wang Q, Zhang Y, Geng M, Wei Y, Liu Y (2020). Multigenerational maternal obesity increases the incidence of HCC in offspring via miR-27a-3p. J Hepatol.

[37] Lee JM, Hammaren HM, Savitski MM, Baek SH (2023). Control of protein stability by post-translational modifications. Nat Commun.

[38] Ciccarone F, Valentini E, Zampieri M, Caiafa P (2015). 5mC-hydroxylase activity is influenced by the PARylation of TET1 enzyme. Oncotarget.

[39] Nakagawa T, Lv L, Nakagawa M, Yu Y, Yu C, D'Alessio AC (2015). CRL4(VprBP) E3 ligase promotes monoubiquitylation and chromatin binding of TET dioxygenases. Mol Cell.

[40] Dawlaty MM, Ganz K, Powell BE, Hu YC, Markoulaki S, Cheng AW (2011). Tet1 Is Dispensable for Maintaining Pluripotency and Its Loss Is Compatible with Embryonic and Postnatal Development. Cell Stem Cell.

[41] Wang Q, Wang W, Zhang A (2021). TET-mediated DNA demethylation plays an important role in arsenic-induced HBE cells oxidative stress via regulating promoter methylation of OGG1 and GSTP1. Toxicol In Vitro.

[42] Wu BK, Mei SC, Chen EH, Zheng Y, Pan D (2022). YAP induces an oncogenic transcriptional program through TET1-mediated epigenetic remodeling in liver growth and tumorigenesis. Nat Genet.

[43] Li X, Zou Y, Fu YY, Xing J, Wang KY, Wan PZ (2021). A-Lipoic Acid Alleviates Folic Acid-Induced Renal Damage Through Inhibition of Ferroptosis. Front Physiol.

[44] Li W, Xiang Z, Xing Y, Li S, Shi S (2022). Mitochondria bridge HIF signaling and ferroptosis blockage in acute kidney injury. Cell Death Dis.

[45] Whaley-Connell AT, Habibi J, Nistala R, DeMarco VG, Pulakat L, Hayden MR (2012). Mineralocorticoid receptor-dependent proximal tubule injury is mediated by a redox-sensitive mTOR/S6K1 pathway. Am J Nephrol.

[46] Yang L, Besschetnova TY, Brooks CR, Shah JV, Bonventre JV (2010). Epithelial cell cycle arrest in G2/M mediates kidney fibrosis after injury. Nat Med.

[47] Nunes DV, Costa CA, De Bem GF, Cordeiro VS, Santos IB, Carvalho LC (2018). Tempol, a superoxide dismutase-mimetic drug, prevents chronic ischemic renal injury in two-kidney, one-clip hypertensive rats. Clin Exp Hypertens.

[48] Ehrlich M (2019). DNA hypermethylation in disease: mechanisms and clinical relevance. Epigenetics-Us.

[49] Wang J, Zhang Y, Zhuo Q, Tseng Y, Wang J, Ma Y (2020). TET1 promotes fatty acid oxidation and inhibits NAFLD progression by hydroxymethylation of PPARalpha promoter. Nutr Metab (Lond).

[50] Huang G, Liu L, Wang H, Gou M, Gong P, Tian C (2020). Tet1 Deficiency Leads to Premature Reproductive Aging by Reducing Spermatogonia Stem Cells and Germ Cell Differentiation. iScience.

[51] Yan H, Tan L, Liu Y, Huang N, Cang J, Wang H (2020). Ten-eleven translocation methyl-cytosine dioxygenase 2 deficiency exacerbates renal ischemia-reperfusion injury. Clin Epigenetics.

[52] Tampe B, Tampe D, Muller CA, Sugimoto H, LeBleu V, Xu X (2014). Tet3-mediated hydroxymethylation of epigenetically silenced genes contributes to bone morphogenic protein 7-induced reversal of kidney fibrosis. J Am Soc Nephrol.

[53] Tampe B, Steinle U, Tampe D, Carstens JL, Korsten P, Zeisberg EM (2017). Low-dose hydralazine prevents fibrosis in a murine model of acute kidney injury-to-chronic kidney disease progression. Kidney Int.

[54] Ciccarone F, Valentini E, Bacalini MG, Zampieri M, Calabrese R, Guastafierro T (2014). Poly(ADP-ribosyl)ation is involved in the epigenetic control of TET1 gene transcription. Oncotarget.

[55] Kim J (2016). Poly(ADP-ribose) polymerase activation induces high mobility group box 1 release from proximal tubular cells during cisplatin nephrotoxicity. Physiol Res.

[56] Devalaraja-Narashimha K, Singaravelu K, Padanilam BJ (2005). Poly(ADP-ribose) polymerase-mediated cell injury in acute renal failure. Pharmacol Res.

[57] Shi FT, Kim H, Lu W, He Q, Liu D, Goodell MA (2013). Ten-eleven translocation 1 (Tet1) is regulated by O-linked N-acetylglucosamine transferase (Ogt) for target gene repression in mouse embryonic stem cells. J Biol Chem.

[58] Xin YJ, Yuan B, Yu B, Wang YQ, Wu JJ, Zhou WH (2015). Tet1-mediated DNA demethylation regulates neuronal cell death induced by oxidative stress. Sci Rep.

[59] Chen Y, Feng X, Hu X, Sha J, Li B, Zhang H (2018). Dexmedetomidine Ameliorates Acute Stress-Induced Kidney Injury by Attenuating Oxidative Stress and Apoptosis through Inhibition of the ROS/JNK Signaling Pathway. Oxid Med Cell Longev.

[60] Shan Y, Chen D, Hu B, Xu G, Li W, Jin Y (2021). Allicin ameliorates renal ischemia/reperfusion injury via inhibition of oxidative stress and inflammation in rats. Biomed Pharmacother.

