# Supplementary material for: Tet1 deficiency exacerbates oxidative stress in acute kidney injury by regulating superoxide dismutase
Source: Theranostics. 2023 Sep 25;13(15):5348–64. doi: 10.7150/thno.87416 (PMC10614682; doi:10.7150/thno.87416)
Supplement: Supplementary file 1 — Supplementary figures and tables. [file thnov13p5348s1.pdf]

Supplemental Information

for

Tet1 deficiency exacerbates oxidative stress in acute kidney injury by regulating superoxide dismutase

Yu Fan[1], Yangmian Yuan[1], Mingrui Xiong[2], Muchuan Jin[1], Donge Zhang[3], Dong Yang[2], Chengyu Liu[4], Robert B. Petersen[5], Kun Huang[2], Anlin Peng[3]*, Ling Zheng[1]*

[1]Hubei Key Laboratory of Cell Homeostasis, Frontier Science Center for Immunology and Metabolism, College of Life Sciences, Wuhan University, Wuhan, China, 430072

[2]School of Pharmacy, Tongji Medical College and State Key Laboratory for Diagnosis and Treatment of Severe Zoonotic Infectious Diseases, Huazhong University of Science and Technology, Wuhan, China, 430030

[3]Department of Pharmacy, The Third Hospital of Wuhan and Tongren Hospital of Wuhan University, Wuhan, China, 430070

[4]Department of Transfusion Medicine, Wuhan Hospital of Traditional Chinese and Western Medicine, Tongji Medical College, Huazhong University of Science and Technology, Wuhan, China, 430030

[5]Foundational Sciences, Central Michigan University College of Medicine, Mount Pleasant, MI, USA. 48858

* Corresponding authors

Ling Zheng, Ph.D.
College of Life Sciences
Wuhan University
Wuhan, China, 430072
E-mail: lzheng@whu.edu.cn

Anlin Peng, Ph.D.
Department of Pharmacy
Tongren Hospital of Wuhan University
Wuhan, China, 430070
E-mail: dr_anlin_peng@163.com

Table S1. Antibodies used in this study.

Antibody	Catalog number	Vendor
Tet1 (WB)	ab191698	Abcam
Bax (WB)	sc-526	Santa Cruz Biotechnology
Bcl2 (WB)	610538	BD Biosciences
Cleaved Caspase 3 (WB, IHC)	9661S	Cell Signaling Technology
Sod1 (WB)	A0274	Abclonal
Sod2 (WB)	13141T	Cell Signaling Technology
3-NT (WB)	MAB5404	EMD Millipore Corporation
Cyclin B1 (WB)	12231	Cell Signaling Technology
Cyclin D1 (WB)	A19038	Abclonal
p-H2A.X (WB)	9718S	Cell Signaling Technology
Hsp90 (WB)	4877S	Cell Signaling Technology
α-Tubulin (WB)	AF0001	Beyotime Biotechnology
Tet1 (IHC, IF, CHIP)	GTX125888	GeneTex
LTL (IF)	FL-1321	Vector Laboratories
PNA (IF)	CL-1073	Vector Laboratories
DBA (IF)	RL-1032	Vector Laboratories
CD3 (IHC)	130914	Gene Tech
Ly6G (IHC)	BD551459	BD Biosciences
F4/80 (IHC)	70076S	Cell Signaling Technology

α-SMA (IHC)	A2547	Sigma
5hmC (IHC, Dot blot)	39770	Active motif
5mC (Dot blot)	ab10805	Abcam
Ki67 (IF)	Ab16667	Abcam
p-H3 (IF)	GTX637402	GeneTex
Tet1 (IP)	GTX124207	GeneTex
PAR/pADPr (WB)	4335-MC-100	R&D SYSTEMS
Ubiquitin (WB)	U5379	Sigma
Rabbit IgG (CHIP, IP)	2729S	Cell Signaling Technology

WB, Western blot; ChIP, Chromatin immunoprecipitation; IF, Immunofluorescence; IHC, Immunohistochemistry.

Table 2. Primers used in the present study.

Gene	Forward	Reverse
**(1) qPCR primer sequences**		
M *Tet1*	GCTGGATTGAAGGAACAGGA	GTCTCCATGAGCTCCCTGAC
M *Tet2*	GACTCAACGGTTATCAGGCTTTT	CATTGCTCTTTATTCTTCCTCTGTAA
M *Tet3*	CTCCCCTGCTGTCTTCAGA	CCTGAGGCTCTGTGGAAGTA
M *Rn18s*	CTCAACACGGGAAACCTCA	CGCTCCACCAACTAAGAACG
M *Kim1*	TCAGATTCAAGTCTTCATTTCAGG	CCCCCTTTACTTCCACATAAGAA
M *Ngal*	CCATCTATGAGCTACAAGAGAACAAT	TCTGATCCAGTAGCGACAGC
M *Ccl9*	GCCCAGATCACACATGCAAC	AGGACAGGCAGCAATCTGAA
M *Ccl6*	TGCCACACAGATCCCATGTA	GTGCTTAGGCACCTCTGAACT
M *Ccl7*	AAGAAGGGCATGGAAGTCTG	TCAAGGCTTTGGAGTTGGG
M *Cxcl5*	GCTGCGTTGTGTTTGCTTAAC	TAGCTATGACTTCCACCGTAGG
M *Sod1*	GGGAAGCATGGCGATGAAAG	CCCCATACTGATGGACGTGG
M *Sod2*	GCCTGCTCTAATCAGGACCC	GTAGTAAGCGTGCTCCCACA
M Hao2	AAGCACAACATCCGAGGCAT	CTAGCTCCAAGGGCTAGTGC
M *Cat*	CACTGACGAGATGGCACACT	TGTGGAGAATCGAACGGCAA
**(2) ChIP primers used in the present study**		
M *Sod1-P1*	GCTGAGGCAGGAGGATCTTA	CCTTTGGCTCTCATGGAACT
M *Sod1-P2*	GCTGATTATGGCACGGATCT	GAGGAAGGAAGACCAACGTG
M *Sod1-P3*	GACAATCCGCATTTCCAGAC	GTGCGGACTGAGAAAGTTCC
M *Sod2-P1*	CACCACACCACCATAGCATT	ACAGTGACACACCCATTCCA
M *Sod2-P2*	CTAGCTGCCTTTGGATGAGG	ACCGGAGCTGTATGGATGAC
M *Sod2-P3*	GATGAACACACGCAAACCTG	CTGGGAAACCCTGGAGACTT
**(3) Primer sequences used for Bisulfite sequencing**		
Sod1	CGGTTAGGGAGTTTTACGAAG	CTTTTATAAACCTAAATCTAACCACGC
Sod2	TTTTTTAGAAGTAGGAGTAGAAATAGAGTG	CCCCTATACCAAATTAATAAAAACC

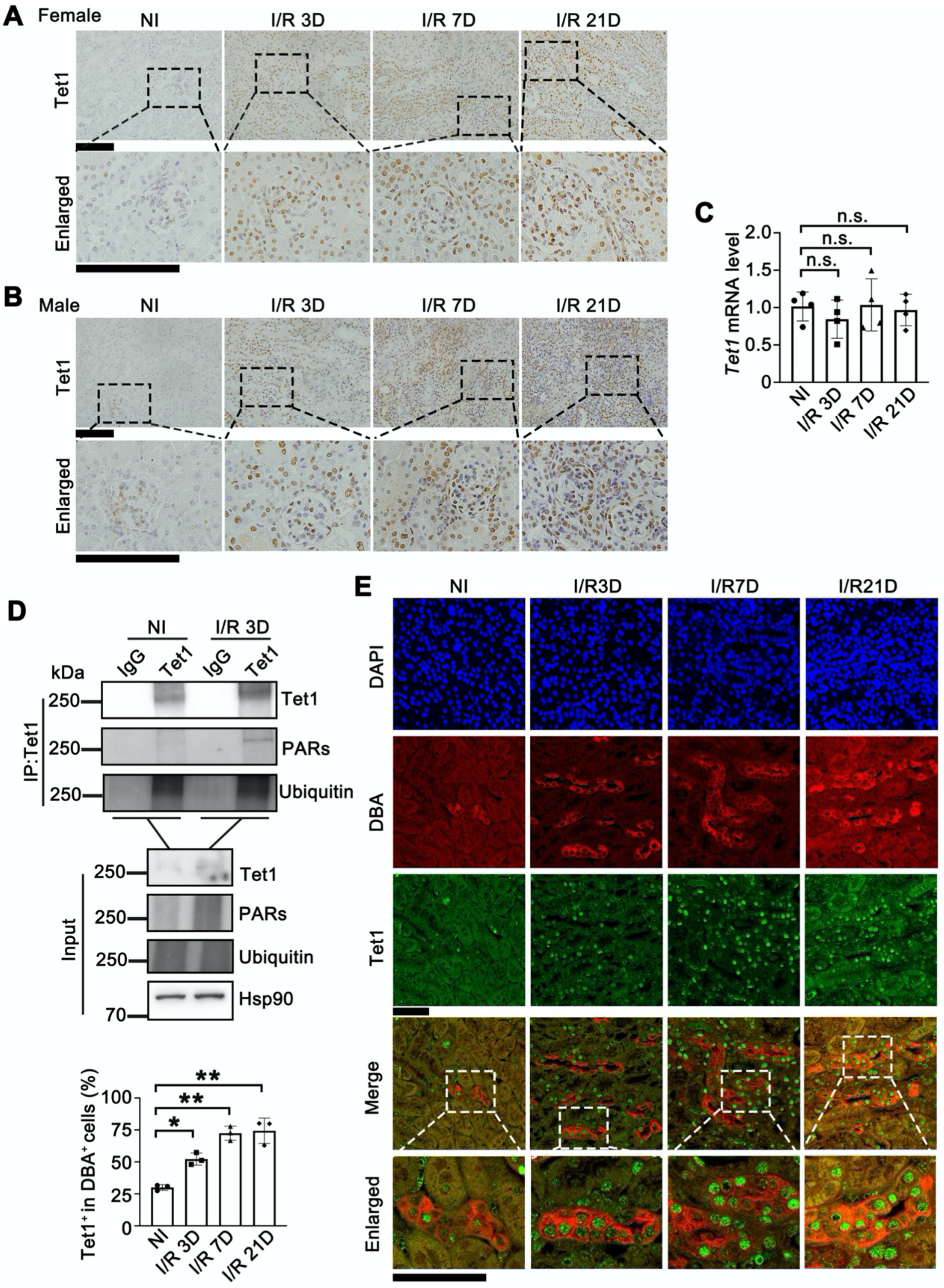

Figure S1. Tet1 is increased in the kidney tubular cells after I/R injury. (**A**) Representative immunochemical staining for Tet1 and quantitative results of the number of Tet1^{+} cells in the kidney of non-injured female mice (NI) or female mice at day 3, 7, or

21 after the I/R injury (I/R 3D, I/R 7D, I/R 21D). Scale bar = 100 μm. (**B**) Representative immunochemical staining for Tet1 in the kidney of non-injured male mice (NI) or male mice at day 3, 7, or 21 after the I/R injury (I/R 3D, I/R 7D, I/R 21D). Scale bar = 100 μm. (**C**) mRNA level of *Tet1* in the kidney of non-injured male mice (NI) or male mice at day 3, 7, or 21 after the I/R injury (I/R 3D, I/R 7D, I/R 21D). (**D**) Co-Immunoprecipitation of Tet1 and PAR polymers (PARs) or Ubiquitin in kidney from female mice with (I/R 3D) or without (NI) renal I/R injury. Co-Immunoprecipitation assays were performed using anti-Tet1 or respective IgG as the negative control. (**E**) Representative co-immunostaining for Tet1 (green) with DBA (dolichos biflorus agglutinin; red) with quantitative results in the kidney of non-injured female mice (NI) or male mice at day 3, 7, or 21 after the I/R injury (I/R 3D, I/R 7D, I/R 21D). DAPI (blue) was used to stain nuclei. Scale bar = 50 μm. *$P <$ 0.05; **$P < 0.01$; n.s. indicates not significant.

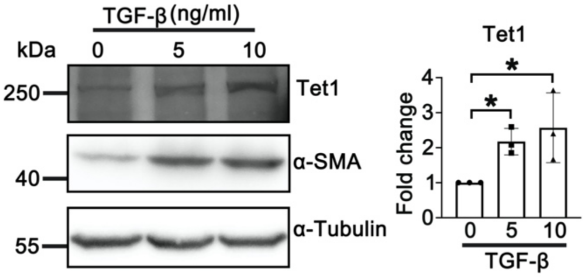

Figure S2. Tet1 is increased in TCMK1 cells after TGF-β treatment. Western blot (left) and quantitative result (right) of Tet1 in TCMK1 cells treated with different concentrations of TGF-β for 24 h. *$P < 0.05$.

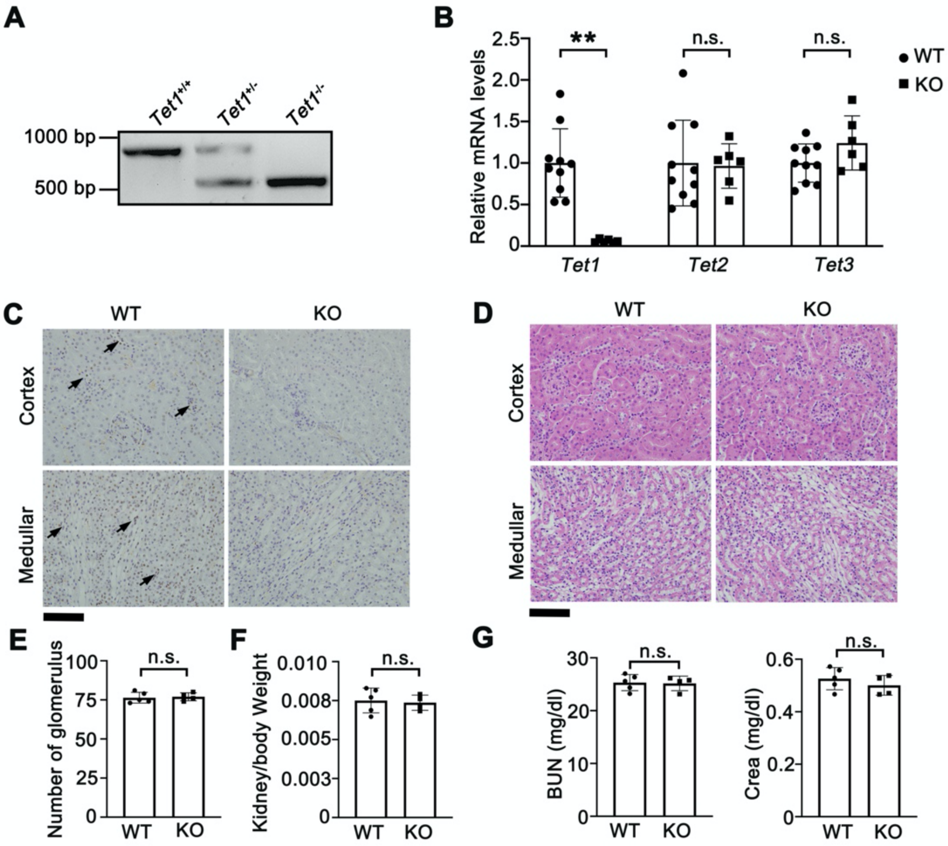

Figure S3. *Tet1* KO female mice show similar renal morphology and function compared to the WT mice. (**A**) Representative genotyping results of *Tet1*$^{+/+}$, *Tet1*$^{+/-}$ and *Tet1*$^{-/-}$ mice. (**B**) mRNA levels of *Tet1*, *Tet2* and *Tet3* in the kidney of WT and *Tet1* KO mice without I/R injury. (**C**) Representative immunochemical images of Tet1 in the kidney of WT and KO mice without I/R injury. Arrows represent Tet1^{+} cell. Scale bar = 100 μm. (**D**) Representative H&E images of the cortex and medulla from WT and KO mice without I/R injury. Scale bar = 100 μm. (**E**) Number of glomeruli in the kidney of WT and *Tet1* KO mice without I/R injury. (**F**) Kidney-to-body weight (B.W.) ratio in the kidney of WT and KO mice without I/R injury. (**G**) BUN (blood urea nitrogen) and Crea (serum creatinine) levels of WT and *Tet1* KO mice without I/R injury. **P < 0.01; n.s. indicates not significant.

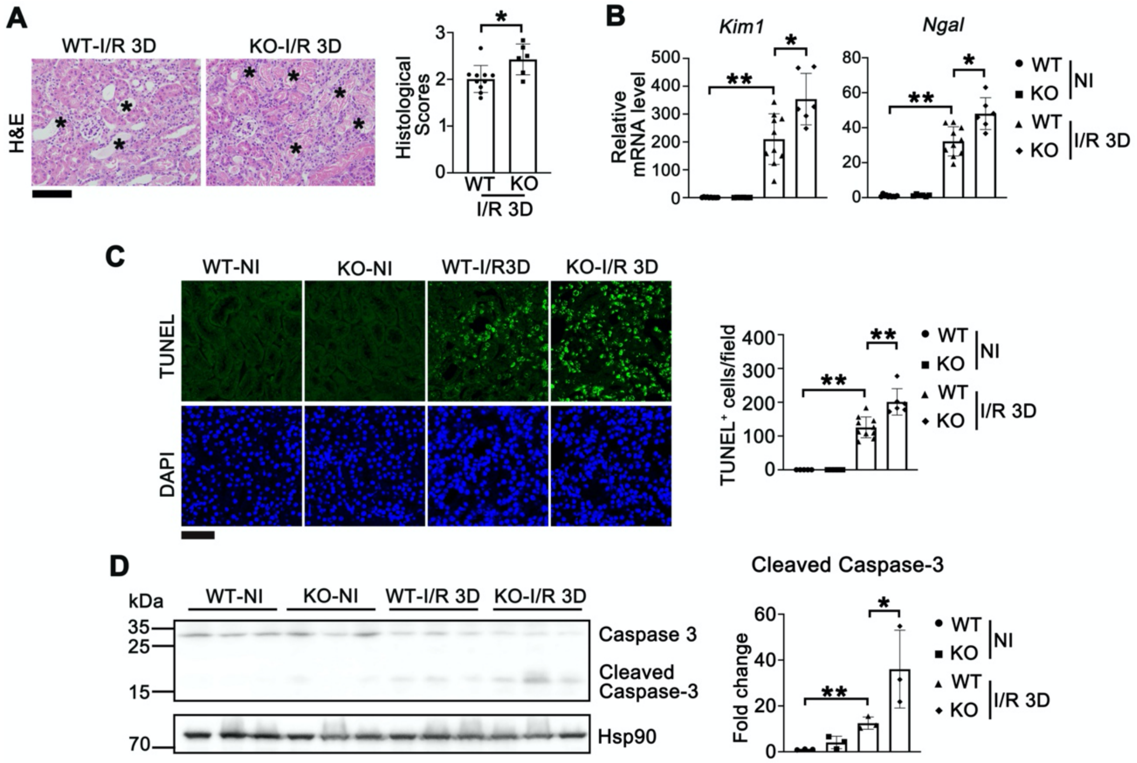

Figure S4. Tet1 knockout increases I/R-induced kidney injury in male mice. (**A**) Representative H&E images with injury scores (right) of the kidney of WT and *Tet1* KO male mice 3 days after the renal I/R injury (I/R 3D). Asterisks indicate injured tubules. Scale bar = 100 μm. (**B**) mRNA levels of *Kim1* and *Ngal* in the kidney of WT and *Tet1* KO male mice with or without injury. (**C**) Representative TUNEL images with quantitative results from WT and *Tet1* KO male mice with or without injury. DAPI was used to stain nuclei. Scale bar = 50 μm. (**D**) Western blots of Caspase 3, cleaved Caspase-3 with quantitative results from the kidney of WT and *Tet1* KO male mice with or without injury. *$P < 0.05$; **$P < 0.01$.

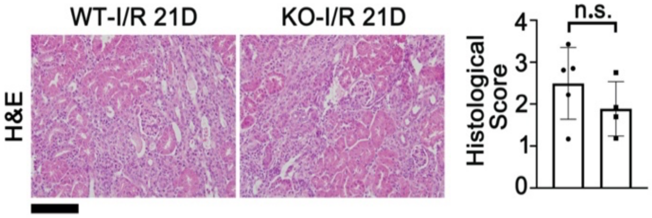

Figure S5. Similar level of pathological injury between *Tet1* KO and WT male mice at I/R 21D. Representative H&E images with injury scores from the kidney of WT and *Tet1* KO male mice at 21 days after the renal I/R injury (I/R 21D). Scale bar = 100 μm. n.s. indicates not significant.

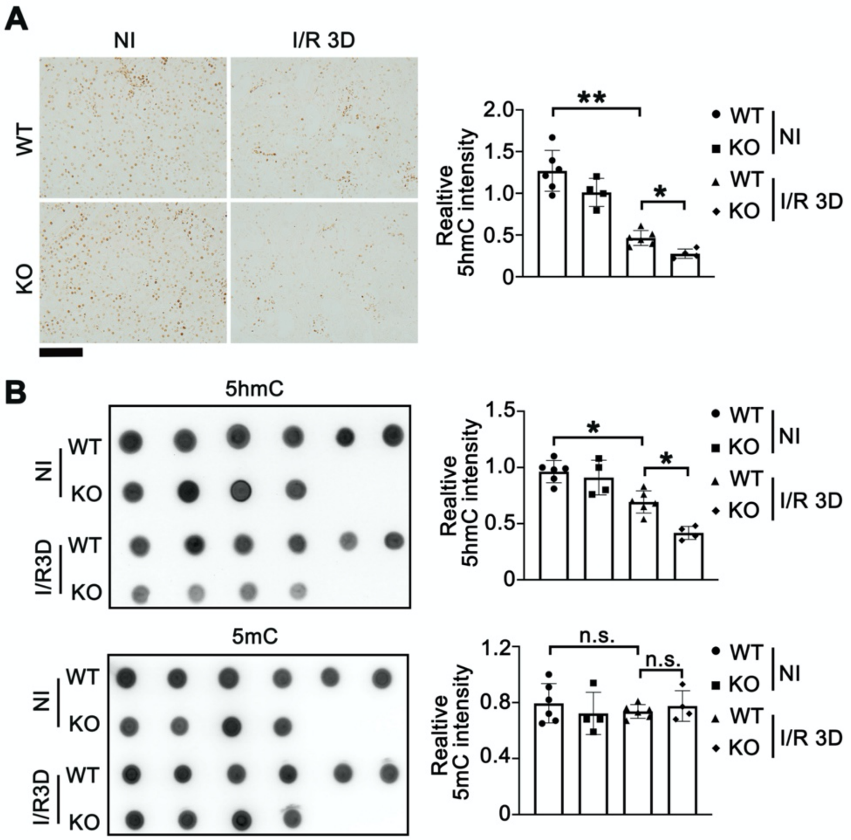

Figure S6. Knockout of Tet1 in female mice further exacerbates the renal 5hmC reduction induced by I/R injury. (**A**) Representative images of immunohistochemistry for 5hmC with quantitative result in the kidney of WT and *Tet1* KO female mice with or without injury. Scale bar = 100 μm. (**B**) Representative dot blot results for anti-5hmC and anti-5mC with quantitative results of the DNA extracted from kidneys of WT and *Tet1* KO female mice with or without injury. *$P < 0.05$; **$P < 0.01$; n.s. indicates not significant.

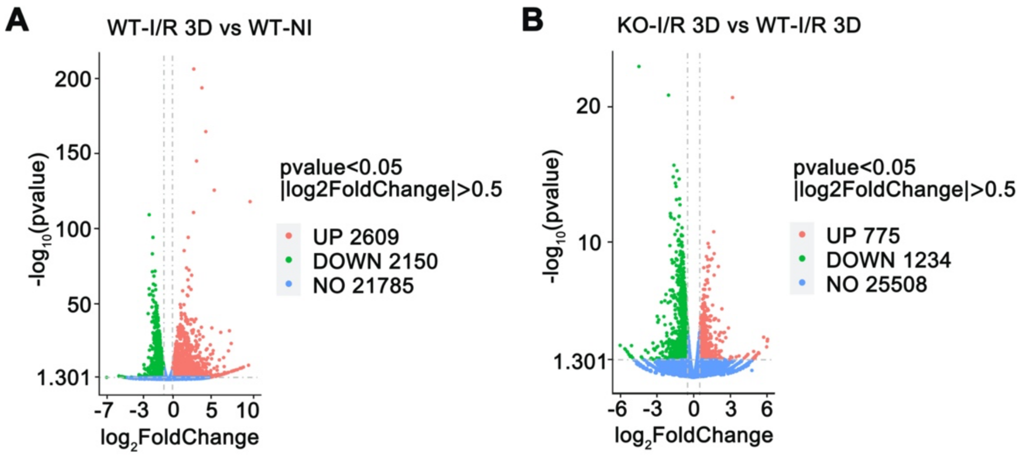

Figure S7. RNA-sequencing analysis of the kidney from WT and *Tet1* KO mice with or without I/R injury. (**A**) Volcanic map showing altered genes in the kidney of WT mice at day 3 after injury (WT-I/R 3D) *vs.* non-injured kidney (WT-NI). (**B**) Volcanic map showing altered genes in the injured kidney from WT (WT-I/R 3D) and *Tet1* KO (KO-I/R 3D) mice.

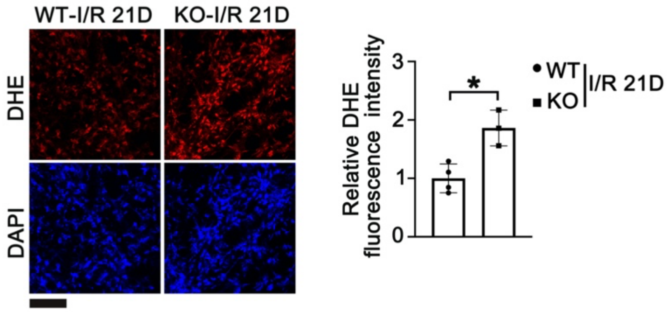

Figure S8. Knockout of Tet1 in female mice shows higher oxidative stress levels at the AKI to CKD stage. Representative DHE staining images with quantitative results from the kidney of WT and *Tet1* KO mice at 21 days after the renal I/R injury (I/R 21D). DAPI was used to stain nuclei. Scale bar = 50 µm.

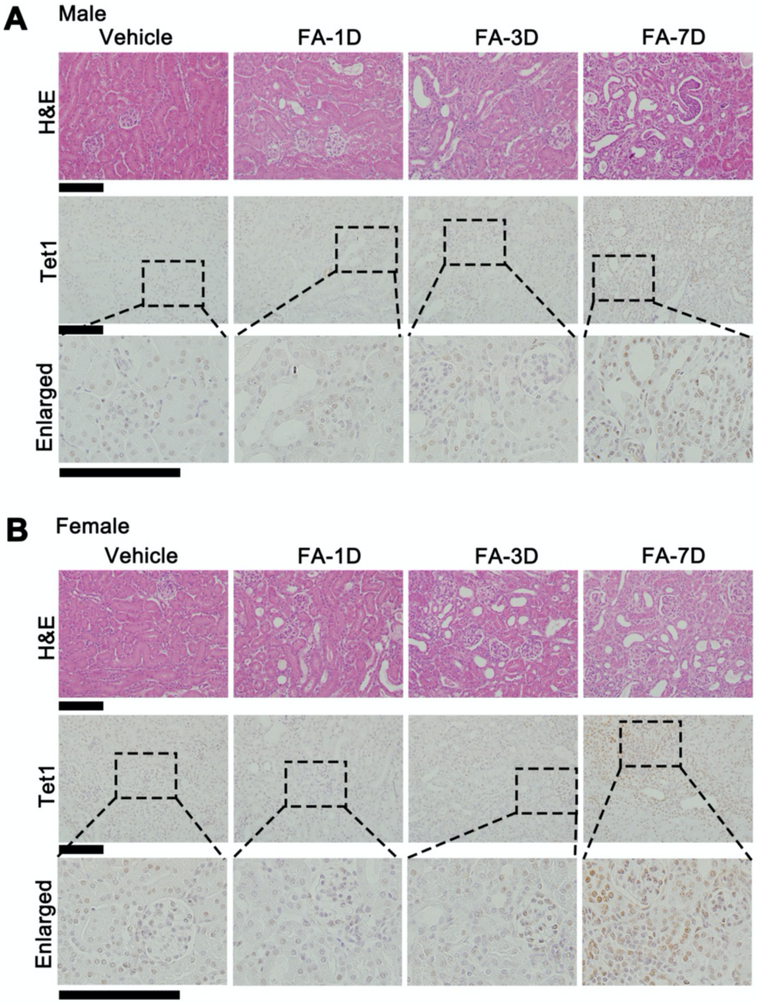

Figure S9. Tet1 responses to folic acid-induced kidney injury in both genders. Representative images of H&E staining and immunostaining for Tet1 in the kidney of male (**A**) and female (**B**) mice at day 1, 3, or 7 after folic acid injection (FA-1D or FA-3D or FA-7D); vehicle injected mice were used as the control (Vehicle). Brown color indicates positive staining. Scale bar = 100 μm.
